# TACC3 enhances glycolysis in bladder cancer cells through inducing acetylation of c-Myc

**DOI:** 10.1038/s41419-025-07645-6

**Published:** 2025-04-17

**Authors:** Zhirui Lin, Falian Liang, Gengde Hong, Xizhen Jiang, Qingling Zhang, Mengyao Wang

**Affiliations:** 1 Institute of Medical Research, Guangdong Provincial People’s Hospital (Guangdong Academy of Medical Sciences), Southern Medical University, Guangzhou, 510080 Guangdong Province People’s Republic of China; 2 Department of Pathology, Guangdong Provincial People’s Hospital (Guangdong Academy of Medical Sciences), Southern Medical University, Guangzhou, 510080 Guangdong Province People’s Republic of China; 3 Radiation Oncology Department, Guangzhou Institute of Cancer Research, the Affiliated Cancer Hospital, Guangzhou Medical University, Guangzhou, 510245 Guangdong Province People’s Republic of China

**Keywords:** Molecular biology, Cancer

## Abstract

The proliferation of bladder cancer (BC) cells is driven by metabolic reprogramming, marked by a glycolytic dependency to sustain uncontrolled growth. While Transforming Acidic Coiled-Coil Containing Protein 3 (TACC3) is known to promote BC progression and correlate with poor prognosis, the mechanisms underlying its upregulation and role in aerobic glycolysis remain unclear. Here, we identify E2F3 as a direct transcriptional activator of *TACC3*, with its amplification in BC driving elevated TACC3 expression. TACC3 overexpression enhances glycolysis, increasing glucose consumption, lactate production, and expression of glycolytic enzymes (e.g., GLUT1, HK2, PFKFB3), while its knockdown suppresses these effects. Pharmacological inhibition of glycolysis abrogates TACC3-driven tumor growth in vitro and in vivo. Mechanistically, TACC3 interacts with c-Myc, promoting its acetylation at lysine 323 (K323) by recruiting the acetyltransferase PCAF and antagonizing the deacetylase SIRT1. This acetylation stabilizes c-Myc, amplifying its transcriptional activation of glycolytic targets. Our findings establish TACC3 as a critical regulator of c-Myc-driven metabolic reprogramming in BC, highlighting its potential as a therapeutic target to disrupt glycolysis and oncogenic c-Myc signaling.

## Introduction

Metabolic reprogramming is a hallmark of cancer [1, 2]. One prominent manifestation of this reprogramming is the increased conversion of glucose to lactate, even under oxygen-rich conditions, a phenomenon known as “aerobic glycolysis” or the “Warburg effect” [3]. This enhanced glycolytic pathway provides cancer cells with sufficient ATP and a diverse array of biomolecules to support their rapid growth and proliferation. Consequently, small-molecule inhibitors targeting glycolytic enzymes have emerged as promising therapeutic strategies [4]. For example, oxamate, a specific lactate dehydrogenase (LDH) inhibitor, enhances the sensitivity of breast cancer cells to taxol [5]. Similarly, 2-deoxy-d-glucose (2-DG), a hexokinase (HK) inhibitor, effectively suppresses tumor growth in vivo with minimal side effects [6].

MYC (c-Myc), a member of the basic helix-loop-helix (HLH) and leucine zipper (LZ) transcription factor family, functions as a potent oncogenic driver [7]. Upon dimerizing with its obligate partner MAX, c-Myc binds to the CACGTG motif to activate the transcription of thousands of target genes involved in critical processes such as cell proliferation, dedifferentiation, anti-apoptotic signaling, and angiogenesis. Notably, c-Myc is a master regulator of metabolic reprogramming, shifting energy production from oxidative phosphorylation (OXPHOS) to aerobic glycolysis (the Warburg effect) [3]. It directly activates the transcription of glycolytic enzymes, including HK1/2, LDHA/B, and pyruvate dehydrogenase kinase (PDK), as well as the glucose transporter GLUT1, thereby promoting glycolysis and suppressing OXPHOS [8]. Furthermore, c-Myc enhances PKM2 expression by activating splicing factors, which reinforces glycolytic flux [9]. Collectively, hyperactive MYC signaling establishes a state of glycolytic dependency in cancer cells, rendering them reliant on this metabolic pathway for survival and growth.

Bladder cancer (BC) remains one of the most common malignancies worldwide and a leading cause of cancer-related mortality in men, with its incidence continuing to rise annually [10]. The standard treatment for advanced BC involves radical cystectomy combined with perioperative cisplatin-based chemotherapy. However, the majority of BC patients eventually develop chemotherapy resistance, posing a significant clinical challenge [11]. Recent studies have identified numerous somatic alterations, including mutations in TERT, FGFR3, TP53, PIK3CA, STAG2, and chromatin modification genes, as well as the FGFR3-TACC3 fusion gene, which have provided critical insights into BC pathogenesis [12, 13]. Despite these advances, the lack of a comprehensive understanding of the molecular mechanisms driving BC progression has hindered the development of effective therapeutic strategies for patients.

Transforming acidic coiled-coil 3 (TACC3), a centrosome- and microtubule-associated protein [14–16], is frequently overexpressed and strongly linked to cancer progression and metastasis across multiple malignancies [17–24]. In bladder cancer (BC), chromosomal translocations can fuse *TACC3* to *FGFR3*, resulting in constitutive activation of FGFR3 kinase and driving cellular transformation [13, 25–27]. Genome-wide association studies (GWAS) further implicate *TACC3* as a candidate susceptibility gene in BC [28, 29]. Previous work from our group demonstrated that TACC3 overexpression correlates with poor prognosis and transcriptionally upregulates *E2F1* to promote proliferation [17]. Nevertheless, the mechanisms driving TACC3 upregulation and its role in BC progression remain poorly understood.

In this study, we demonstrate that E2F3 directly activates the transcription of *TACC3* in bladder cancer. Importantly, we reveal that TACC3 plays a critical role in regulating glycolysis, primarily through MYC-driven transcriptional activation. Furthermore, we show that TACC3 physically interacts with MYC and enhances its acetylation, thereby stabilizing MYC and amplifying its oncogenic activity. Collectively, our findings establish TACC3 as a key regulator of glycolysis and a promising therapeutic target in BC.

## Results

### TACC3 is an E2F3 target gene

**Fig. 1:**
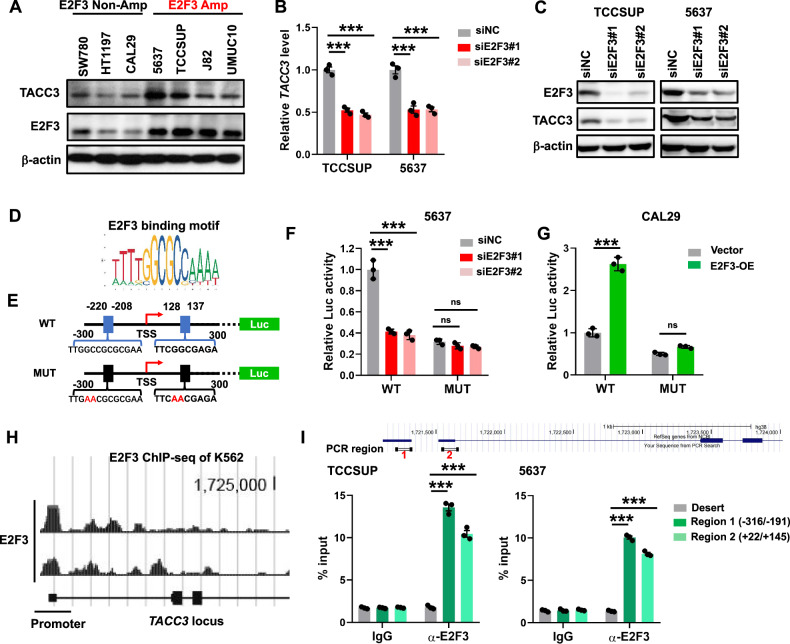
TACC3 is a direct target of E2F3. **A** WB analysis of TACC3 protein expression in a panel of bladder cancer cell lines with or without E2F3 amplification. **B** QPCR analysis of *TACC3* mRNA levels in TCCSUP and 5637 cells transfected with two specific E2F3 siRNAs or negative control siNC. **C** WB analysis of indicated protein in siE2F3-transfected TCCSUP and 5637 cells. **D** Predicted E2F3 binding motifs (JASPAR ID: MA0469.3) identified using the JASPAR database. **E** Schematic diagram of the constructed luciferase reporters containing wild-type (WT) or mutant (MUT) TACC3 promoter sequences. The transcription start site (TSS) is marked at position 0. Two predicted E2F3 binding sites are located at positions −220 to −208 (Site 1) and +128 to +137 (Site 2) relative to the TSS. Mutations in both sites (red Xs) were introduced into the promoter fragments. **F** Dual-luciferase reporter assays in 5637 cells co-transfected with WT or mutant *TACC3* promoter reporters and siNC or *E2F3* siRNAs. **G** Dual-luciferase reporter assays in CAL29 cells co-transfected with WT or mutant *TACC3* promoter reporters and an empty vector or *E2F3* overexpression plasmid. **H** ChIP-seq data mapping showing E2F3 enrichment at *TACC3* promoter regions in K562 cells. **I** Upper, Schematic of the *TACC3* genomic locus with PCR amplification regions. Lower, ChIP-qPCR assays showing E2F3 binding at the *TACC3* promoter (−316/−191) and leader (+22/+145) regions. IgG served as a negative control. All the experiments have been repeated in triplicate. Statistical significance was calculated by **B**, **E**, **H** one-way ANOVA; **F** two-tailed student’s *t*-test; *n* = 3 biological repeats. Data were presented as means ± SD. ***P < 0.001.

Transcription factors (TFs) are known to regulate downstream co-expressed genes within biological networks [30]. To identify upstream regulators of *TACC3*, we first analyzed genes positively associated with *TACC3* mRNA levels using mRNA expression profiles from 34 bladder cancer cell lines in the DepMap (23Q4) database [31] (Fig. S1A). Transcription factor enrichment analysis revealed that E2F family proteins were among the top preferentially enriched transcription factors (Fig. S1B). Among the E2F family activators, we focused on E2F Transcription Factor 3 (E2F3) because *E2F3* gene amplification occurs in ~9% of BC cases [32]. This amplification may represent a lineage-specific event in BC and is associated with more advanced disease stages [33]. We observed a positive correlation between *TACC3* mRNA expression and *E2F3* mRNA expression in BC cell lines (Fig. S1C). Furthermore, 12 out of 34 bladder cancer cell lines with *E2F3* amplification, defined as log2(copy number) ≥1.3, exhibited higher levels of *TACC3* mRNA expression (Fig. S1D and Table S1). To validate these findings, we confirmed that *E2F3*-amplified BC cell lines showed significantly higher E2F3 protein levels compared to non-amplified lines. Consistent with this, TACC3 protein expression was markedly elevated in *E2F3*-amplified cells (Fig. 1A). To assess clinical relevance, we analyzed mRNA profiles from The Cancer Genome Atlas (TCGA) BC cohort and found that *TACC3* mRNA levels positively correlated with *E2F3* copy number status (Fig. S1E). These findings suggest that *TACC3* is a target of E2F3 during BC progression.

To determine whether E2F3 directly regulates TACC3 transcription, we silenced E2F3 via shRNAs in TCCSUP and 5637 cell lines and observed a significant reduction in both TACC3 mRNA and protein levels (Fig. 1B, C). Conversely, E2F3 overexpression in T24 and CAL29 cell lines led to a marked increase in TACC3 expression (Fig. S1F–G). Using the JASPAR database, we analyzed the genomic sequence of *TACC3* and identified two candidate E2F3-binding sites with a highly conserved sequence, TTTSSSCG (where S denotes a purine base) (Fig. 1D). One site was located within the promoter region (~220 to ~208 upstream of the transcription start site), and the other was downstream in the leader sequence (+128 to +137). We constructed luciferase reporters containing wild-type or mutated E2F3-binding sites (Fig. 1E). and performed luciferase reporter assays. Depletion of *E2F3* suppressed luciferase activity driven by the *TACC3* promoter, while *E2F3* overexpression enhanced *TACC3* promoter activity. Mutating both binding sites simultaneously abolished *E2F3*-mediated transcriptional activation and eliminated *TACC3* promoter-driven luciferase activity (Fig. 1F, G). Importantly, we identified notable E2F3 binding peaks in the *TACC3* promoter regions using chromatin immunoprecipitation followed by deep sequencing (ChIP-seq) data from K562 cells in the ENCODE project [34] (Fig. 1H). We further confirmed E2F3 binding to the *TACC3* genomic regulatory regions using ChIP–quantitative polymerase chain reaction (qPCR) assays in two BC cell lines (Fig. 1I). Notably, ChIP-seq data from ENCODE also showed E2F1 binding at these loci (Fig. S1H), and ChIP-qPCR validated E2F1 enrichment at the same *TACC3* regulatory regions in BC cells (Fig. S1I), consistent with the overlapping DNA-binding preferences of E2F family members. Collectively, these results demonstrate that E2F3 binds to E2F response elements (E2F-REs) within the genomic regulatory region of *TACC3* and directly activates its transcription.

### TACC3 promotes aerobic glycolysis

**Fig. 2:**
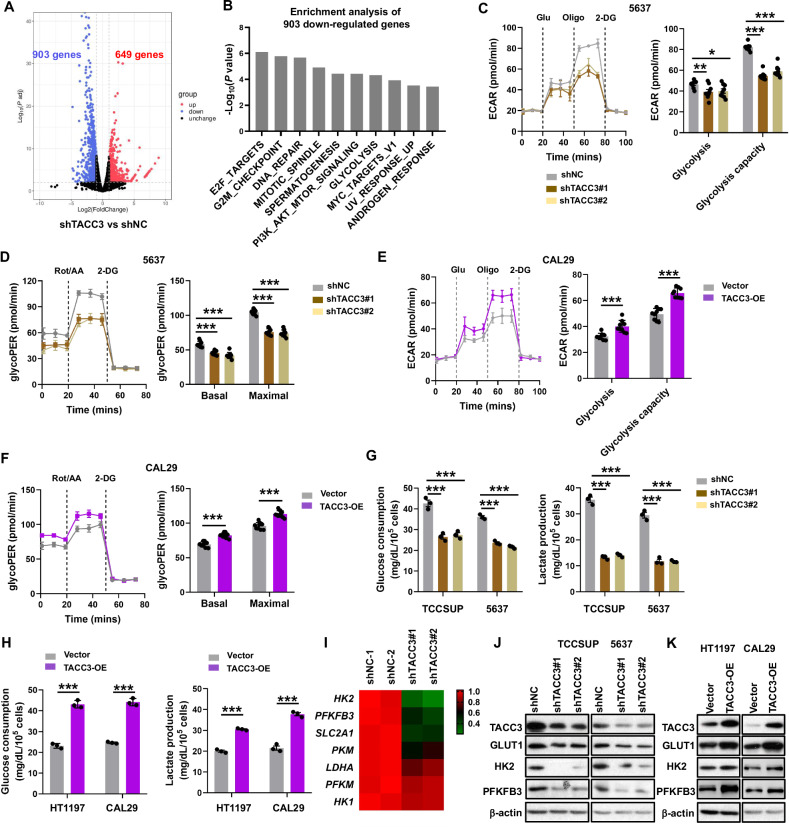
TACC3 enhanced glucose metabolism reprogramming in BC cells. **A** Volcano plot shows differentially expressed genes in 5637 cells with stable TACC3 knockdown compared to control cells. **B** The top 15 enriched HALLMARK gene sets of 903 downregulated genes. **C** Left, ECAR was determined in 5637 cells with or without TACC3 depletion using a Seahorse XF96 Extracellular Flux analyzer. Glu glucose, Oligo oligomycin. Right, statistical analysis of the effects of TACC3 knockdown on glycolytic activity. **D** ECAR was quantified in CAL29 cells with or without TACC3 overexpression as described in (**C**). **E** Analyzes of glycolytic proton efflux rate (glycoPER) of TACC3 knockdown cells using a Seahorse extracellular flux analyzer. **F** Analyzes of glycolytic proton efflux rate (glycoPER) of TACC3 overexpressing cells using a Seahorse extracellular flux analyzer. **G** Glucose consumption and lactate production of TCCSUP and 5637 cells with TACC3 knockdown compared to control cells. **H** Glucose consumption and lactate production of HT1197 and CAL29 cells with TACC3 overexpression compared to control cells. **I** Gene expression heat map showed the comparison of the mRNA level of key glycolysis enzymes between TACC3 knockdown cells and control cells. **J** WB analysis of indicated glycolytic gene in TACC3 knockdown cell lines. **K** WB analysis of indicated glycolytic gene in TACC3 overexpressed cell lines. All the experiments have been repeated in triplicate. Statistical significance was determined by **C**, **D** one-way ANOVA; **E**, **F** two-tailed Student’s *t*-test; *n* = 3 biological repeats. Data were presented as means ± SD. ***P < 0.001.

To investigate the molecular mechanisms by which TACC3 promotes malignant phenotypes in bladder cancer, we performed RNA-seq analysis of 5637 cells with stable *TACC3* knockdown compared to control cells (Fig. 2A). Gene Set Enrichment Analysis (GSEA) [35] revealed that the top ten HALLMARK pathways inhibited in TACC3 knockdown cells included E2F targets, G2M checkpoint, DNA replication, mitotic spindle, spermatogenesis, PI3K-AKT-mTOR signaling, glycolysis, MYC targets V1, UV response up, and androgen response (Fig. 2B). While prior studies have implicated TACC3 in cell cycle regulation and AKT signaling [17, 36–39], its role in glycolysis remained unexplored. Given the therapeutic relevance of metabolic reprogramming in cancer, we focused on elucidating TACC3’s impact on glycolytic metabolism. TACC3-knockdown 5637 cells exhibited reduced extracellular acidification rate (ECAR) and glycolytic proton efflux rate (glycoPER) (Fig. 2C, D), alongside a significant increase in oxygen consumption rate (OCR) (Fig. S2A), compared to the shNC control group. Conversely, TACC3 overexpression in CAL29 cells elevated ECAR and glycoPER levels (Fig. 2E, F) while reducing OCR (Fig. S2B). Consistent with these findings, TACC3 knockdown suppressed glucose consumption and lactate production, whereas TACC3 overexpression enhanced these metabolic activities in BC cell lines (Fig. 2G, H). RNA-seq data further demonstrated that key glycolytic enzymes, including *GLUT1*, *HK2*, and *PFKFB3*, were significantly downregulated (fold change ≤0.5) in TACC3-knockdown cells (Fig. 2I). Western blot analysis confirmed reduced protein levels of these enzymes in TACC3-knockdown cells and increased levels in TACC3-overexpressing cells (Fig. 2J, K). Additionally, single-sample GSEA (ssGSEA) scores for glycolysis positively correlated with TACC3 expression in BC cell lines and tumor samples (Fig. S2C, D). Together, these findings demonstrate that TACC3 enhances glycolysis in bladder cancer.

### Inhibition of the glycolysis pathway suppresses TACC3-induced cell proliferation

**Fig. 3:**
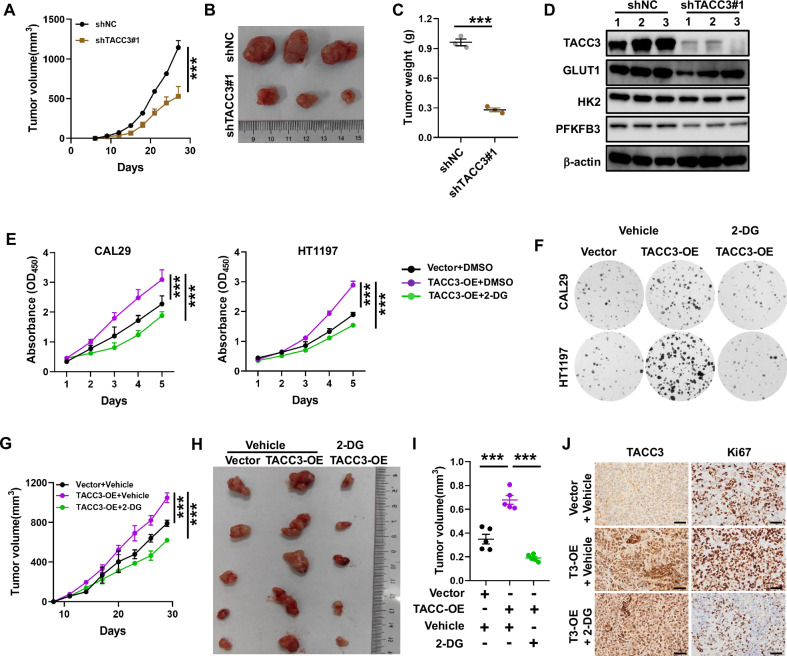
TACC3 enhanced tumor growth and aerobic glycolysis in vivo*.* **A**–**C** Xenograft tumor growth of TACC3 knockdown BC 5637 stable cell lines in nude mice. Tumor size (**B**) and tumor weight (**C**) of two groups were measured and presented. *n* = 3 mice per group. **D** Expression of indicated glycolysis proteins were detected in xenografts by immunoblotting. 1, 2, and 3 represent tumor tissues from distinct mice. **E**, **F** Cell proliferation (**E**) and colony formation assays (**F**) in CAL29 or HT1197 cells with indicated treatment. 2-DG (12.5 mg/ml). Experiments have repeated at least triplicate. **G**–**I**. Xenograft tumor growth of TACC3 overexpression CAL29 cells in nude mice, followed by treatment with intraperitoneal injection of PBS or 2-DG. Tumor size (**H**) and tumor weight (**I**) of three groups were measured and presented. *n* = 5 mice per group. **J** Representative images of TACC3, and Ki67 staining in xenografts of indicated groups in (**H**). Scale bars, 50 μm.

To evaluate the effect of TACC3 on bladder cancer (BC) growth in vivo, we established a xenograft model by subcutaneously injecting TACC3-knockdown 5637 cells and control cells into mice. Tumors derived from TACC3-knockdown cells exhibited significantly smaller volumes and weights compared to control tumors (Fig. 3A–C). The immunoblotting analysis further revealed that TACC3 depletion reduced the expression of glycolysis-related proteins, including GLUT1, HK2, and PFKFB3 (Fig. 3D). To determine whether TACC3 drives BC cell growth through glycolysis, we performed in vitro assays using the hexokinase inhibitor 2-DG. Treatment with 2-DG abolished the pro-growth and colony-forming effects of TACC3 overexpression (Fig. 3E, F). Similar results were observed with another hexokinase inhibitor, 3-BP (Fig. S3A–B). In in vivo xenograft assays, TACC3 overexpression significantly enhanced tumor growth, while 2-DG treatment attenuated this effect (Fig. 3G–J). Collectively, these findings demonstrate that TACC3 promotes tumor growth and glycolysis, and its oncogenic effects can be partially reversed by glycolysis inhibitors.

### TACC3 promoted aerobic glycolysis dependent on c-Myc

**Fig. 4:**
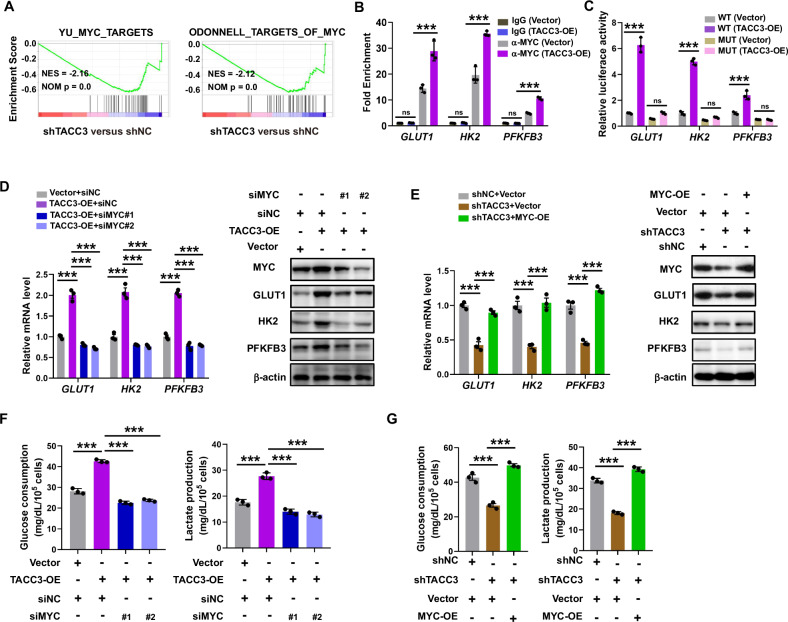
The functional role of TACC3 on aerobic glycolysis is dependent on c-Myc. **A** GSEA result showed the enrichment of MYC target genes is negatively associated with TACC3 depletion. **B** ChIP-qPCR analysis of c-Myc binding at the promoter of GLUT1, HK2, and PFKFB3 gene loci in TACC3-overexpressed CAL29 cells using IgG or anti-Myc antibodies. **C** Dual-luciferase reporter assay in TACC3-overexpressed CAL29 cells transfected with indicated glycolytic gene promoter reporters with c-Myc binding site wild-type or mutant. WT wild-type, MUT mutant. **D** Western blot analysis of the expression of indicated proteins in control or TACC3-overexpressed CAL29 cells transfected with indicated siRNAs. **E** Western blot analysis of the expression of indicated proteins in control or TACC3-knockdown 5637 cells transfected with indicated plasmids. **F** MYC depletion reverses the effects of TACC3 overexpression on glucose consumption and lactate release in CAL29 cells. **G** MYC overexpression reverses the effects of TACC3 knockdown on glucose consumption and lactate production in 5637 cells. All the experiments have been repeated in triplicate. Statistical significance was determined by **B**, **C** two-tailed Student’s *t*-test; **D**–**G** one-way ANOVA; *n* = 3 biological repeats. Data were presented as means ± SEM. ***P < 0.001, ns not significantly.

To elucidate the mechanism by which TACC3 enhances aerobic glycolysis, we analyzed RNA-seq data and found that TACC3 knockdown reduced the enrichment of MYC target genes (Fig. 2A). Two additional MYC target gene sets were also negatively enriched in TACC3-knockdown cells compared to controls (Fig. 4A). We next performed ChIP-qPCR assays in TACC3-overexpressing BC cell lines, which revealed that TACC3 overexpression significantly increased c-Myc binding to the promoter regions of *GLUT1*, *HK2*, and *PFKFB3* (Fig. 4B). Luciferase reporter assays further demonstrated that TACC3 overexpression enhanced the promoter activity of these glycolytic genes, while mutations in the c-Myc binding sites abolished this effect (Fig. 4C). Quantitative reverse transcription PCR (qRT-PCR) and Western blot (WB) assays showed that the upregulation of GLUT1, HK2, and PFKFB3 induced by TACC3 overexpression was reversed by c-Myc knockdown (Fig. 4D). Conversely, c-Myc overexpression rescued the downregulation of these genes caused by TACC3 depletion (Fig. 4E). Functional rescue experiments confirmed that MYC knockdown abolished the pro-glycolytic effects of TACC3 overexpression, as evidenced by reduced glucose consumption and lactate release. Similarly, MYC overexpression reversed the inhibitory effects of TACC3 knockdown on these glycolytic phenotypes (Fig. 4F, G). Collectively, these findings demonstrate that TACC3 enhances aerobic glycolysis by promoting c-Myc-mediated transcriptional activation of key glycolytic genes.

### TACC3 interacts with c-Myc and regulates its transcriptional activity

**Fig. 5:**
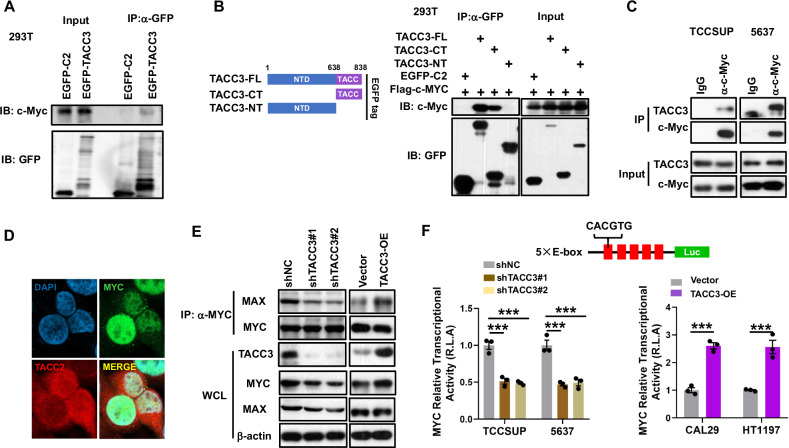
TACC3 interacts with c-Myc. **A** Co-immunoprecipitation (Co-IP) identified the interaction between TACC3 and c-Myc. **B** Mapping of TACC3 protein regions interacting with c-Myc. HEK293T cells were co-transfected with the indicated plasmids of TACC3 (EGFP-tagged) and c-Myc (Flag-tagged). Cell lysates were IP with an anti-GFP. **C** The endogenous interaction between TACC3 and c-Myc in TCCSUP and 5637 cells was evaluated by IP assays using c-Myc antibody. IgG indicates the negative control. **D** Co-localization of c-Myc /TACC3 was detected in 5637 cells with immunostaining. Scale bar, 10 μm. **E** Effects of the TACC3 knockdown or overexpression on the interaction of c-Myc with MAX shown by IP assays using indicated antibodies. **F** Upper, schematic diagram of the constructed luciferase plasmid containing five tandem E-box elements. Lower, dual-luciferase reporter assay using 5× E-box luciferase reporter showed effects of the TACC3 knockdown (Left) or overexpression (Right) on c-Myc transcriptional activity in indicated cell lines. Statistical significance was determined by (Left) one-way ANOVA; (Right) two-tailed Student’s *t*-test; *n* = 3 biological repeats. Data were presented as means ± SD. ***P < 0.001. All the experiments have been repeated in triplicate.

To investigate the potential interaction between TACC3 and c-Myc, we performed a co-immunoprecipitation (Co-IP) assay. The result confirmed an interaction between exogenously expressed EGFP-TACC3 and endogenous c-Myc in 293 T cells (Fig. 5A). Domain mapping revealed that the TACC domain of TACC3 mediates this interaction (Fig. 5B). Furthermore, endogenous TACC3 interacted with c-Myc in TCCSUP and 5637 BC cells (Fig. 5C). Immunofluorescence analysis demonstrated the co-localization of TACC3 and c-Myc in the nucleus of 5637 cells (Fig. 5D), providing additional evidence for their interaction.

c-Myc dimerizes with MAX to activate transcription by binding to E-box sequences (CACGTG), a process critical for both physiological and pathological functions [40]. We found that TACC3 depletion enhanced the interaction between c-Myc and MAX, while TACC3 overexpression impaired this interaction (Fig. 5E). To assess whether TACC3 regulates c-Myc transcriptional activity, we performed luciferase reporter assays using a construct containing five tandem repeats of the c-Myc/MAX binding motif (CACGTG, E-box). TACC3 overexpression significantly increased c-Myc transcriptional activity, whereas TACC3 knockdown reduced it (Fig. 5F). These findings demonstrate that TACC3 interacts with c-Myc, modulates its binding to MAX, and enhances its transcriptional activity.

### TACC3 promotes the acetylation of c-Myc

**Fig. 6:**
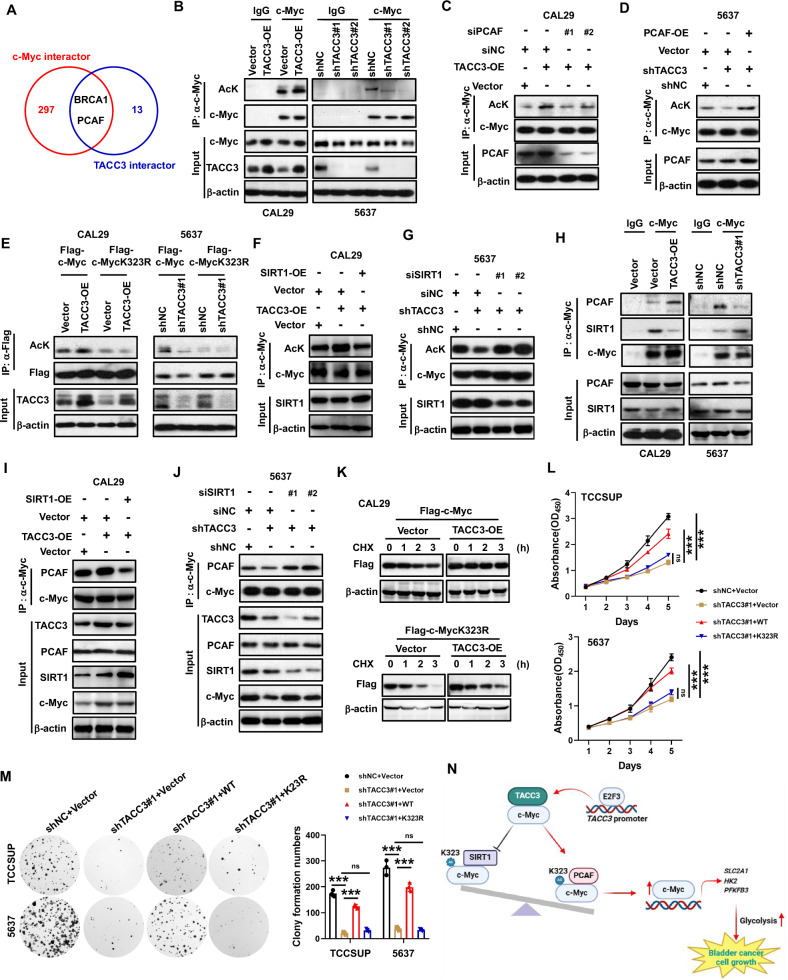
TACC3 stabilizes c-Myc through enhancing c-Myc acetylation at the K323 site. **A** Venn diagram showing overlapping interactors of TACC3 and c-Myc identified via BioGRID (see Methods for database parameters and filtering criteria). **B** The effects of TACC3 deletion or overexpression on the acetylation of c-Myc were assessed by Co-IP. **C** Effect of PCAF silencing on acetylation of c-Myc in CAL29 cells with TACC3 overexpression or control cells. Indicated cells were transfected with the indicated siRNAs for 24 h. Cell extracts were IP with an anti-MYC Ab. Acetylated MYC was detected by immunoblotting. **D** Effect of PCAF overexpression on acetylation of c-Myc in 5637 cells with TACC3 knockdown or control cells. **E** The indicated constructs were transfected into control and TACC3 overexpression or knockdown cells and respective control cells. 48 h later, cell extracts were subjected to immunoprecipitation and immunoblotting with the indicated antibodies. To compare the acetylation of c-Myc, the levels of c-Myc were equalized. **F** Effect of SIRT1 overexpression on acetylation of c-Myc in CAL29 cells with TACC3 overexpressing or control cells. **G** Effect of SIRT1 silencing on acetylation of c-Myc in 5637 cells with TACC3 knockdown or control cells. **H** Effects of the TACC3 overexpression or depletion on the interaction of MYC with PCAF and SIRT1. **I** Effects of the SIRT1 overexpression on the interaction of MYC with PCAF in TACC3 overexpression stable cells. **J** Effects of the SIRT1 knockdown on the interaction of MYC with PCAF in TACC3 knockdown stable cells. **K** The indicated plasmids were transfected into CAL29 cells with TACC3 overexpression and control cells. 36 h later, cells were treated with 0.1 mg/ml cycloheximide (CHX) and harvested at the indicated time. Immunoblots of c-Myc and β-actin protein at the indicated times are shown. **L** CCK-8 assays showed the effect of the MYC WT or K323R mutation on the proliferation of TCCSUP or 5637 cells with or without TACC3 depletion. **M** Effect of the MYC WT or K323R mutation on the colony-forming ability of TCCSUP or 5637 cells with or without TACC3 depletion. Left, representative results are from three independent experiments. Right, statistics of colony number. Data are presented as means ± SD. Statistical significance was determined by one-way ANOVA; *n* = 3 biological repeats. ***P < 0.001, ns not significant. **N** Schematic diagram of TACC3 functional and mechanism in BCs. All the experiments have been repeated in triplicate.

To further elucidate the mechanism by which TACC3 regulates c-Myc transcriptional activity, we analyzed overlapping interactors of TACC3 and c-Myc using the BioGRID database [41, 42], identifying BRCA1 and histone acetyltransferase KAT2B (PCAF) as shared binding partners (Fig. 6A). Given the critical role of acetylation in modulating c-Myc activity, we focused on PCAF, which directly acetylates c-Myc [43]. We hypothesized that TACC3 regulates c-Myc acetylation through PCAF. Indeed, TACC3 overexpression increased c-Myc acetylation, while TACC3 knockdown reduced it (Fig. 6B). Furthermore, the decrease in c-Myc acetylation in TACC3-depleted BC cells was rescued by PCAF overexpression, whereas PCAF silencing reversed the hyperacetylation induced by TACC3 overexpression (Fig. 6C, D). These results demonstrate that TACC3 promotes c-Myc acetylation through PCAF recruitment.

It was reported that lysine 323 (K323) of c-Myc is a major PCAF acetylation site [43]. To validate this, we generated a K323R mutation (replacing lysine with non-acetylate arginine) in c-Myc and measured acetylation levels in TACC3-overexpressing CAL29 cells. The c-Myc K323R mutant exhibited reduced acetylation compared to wild-type c-Myc and diminished the effect of TACC3 overexpression on c-Myc acetylation. Additionally, TACC3 knockdown failed to reduce acetylation of the c-Myc K323R mutant, unlike wild-type c-Myc (Fig. 6E). These results demonstrate that TACC3 induces c-Myc acetylation specifically at K323. Since SIRT1 deacetylates c-Myc at K323 [44], we modulated SIRT1 levels and observed that SIRT1 overexpression suppressed TACC3-mediated hyperacetylation, while SIRT1 silencing restored c-Myc acetylation in TACC3-depleted cells (Fig. 6F, G). These results establish that TACC3 regulates c-Myc acetylation by antagonizing SIRT1.

We further observed that TACC3 overexpression enhanced the interaction between c-Myc and PCAF while reducing c-Myc binding to SIRT1. Conversely, TACC3 knockdown diminished PCAF-c-Myc association and increased SIRT1-c-Myc interaction (Fig. 6H). Furthermore, SIRT1 overexpression disrupted the TACC3-PCAF-c-Myc complex, whereas SIRT1 knockdown strengthened this interaction (Fig. 6I, J), demonstrating competitive binding between TACC3-PCAF and SIRT1 for c-Myc.

Previous studies indicate that acetylation stabilizes c-Myc [43, 45–47]. To test whether TACC3 regulates c-Myc stability, cycloheximide (CHX) chase assays were conducted and revealed that TACC3 overexpression increased the stability of wild-type (WT) c-Myc but had no effect on the c-Myc K323R mutant, which exhibited lower basal stability than WT c-Myc (Fig. 6K). This confirms that TACC3 stabilizes c-Myc via K323 acetylation. Functional rescue experiments further demonstrated that WT c-Myc, but not the K323R mutant, restored proliferation and colony formation in TACC3-depleted cells (Fig. 6L, M). In summary, our findings demonstrate that TACC3-PCAF competes with SIRT1 to bind c-Myc, enhancing its acetylation at K323 and thereby stabilizing c-Myc to promote colony formation and proliferation in bladder cancer.

## Discussion

Although emerging evidence highlights the strong prognostic value of elevated TACC3 expression across various cancers and its potential as a therapeutic target [23, 24], the molecular mechanisms driving TACC3 upregulation remain poorly understood. Here, we demonstrate for the first time that E2F3 directly binds to the *TACC3* promoter and activates its transcription. Supporting its oncogenic role, *E2F3* is focally amplified in bladder cancer, a genomic alteration associated with advanced disease and poor clinical outcomes. While our study primarily focused on E2F3-mediated regulation, we also observed significant enrichment of E2F1 at the *TACC3* promoter (Fig. S1H). Notably, we previously reported that TACC3 transcriptionally upregulates *E2F1* [17]. Future studies should investigate a potential cross-regulatory loop between E2F family members [48] and TACC3, which could create a feedforward or feedback mechanism that amplifies oncogenic signaling in bladder cancer. Additionally, other transcription factor (TF) motifs (e.g., SP1, AP1, NF-κB) were identified in the *TACC3* regulatory regions, warranting future exploration of their interplay with E2F3 in fine-tuning *TACC3* transcription.

Cancer cells are often addicted to glycolysis, which provides substrates for the synthesis of biomolecules essential for rapid growth and survival. Our work demonstrates that TACC3 overexpression promotes aerobic glycolysis in bladder cancer (BC) cells, accompanied by the upregulation of glycolytic genes. Conversely, *TACC3* depletion suppresses glycolysis in both BC cells and xenografts, highlighting its role in reprogramming glucose metabolism. Pharmacologic inhibition of glycolysis reversed the pro-proliferative effects of TACC3 overexpression, indicating that TACC3-driven BC cells are dependent on glycolysis. Furthermore, the correlation between *TACC3* expression and glycolytic activity in human BC specimens corroborates our in vitro findings. Notably, RNA-seq data revealed reduced PKM expression in TACC3-knockdown cells. Further validation showed that TACC3 silencing upregulated PKM1 but downregulated PKM2, while TACC3 overexpression had the opposite effect (Data not shown). These findings suggest that TACC3 regulates glycolysis through both c-Myc-dependent (direct transcriptional activation) and -independent (e.g., PKM splicing) mechanisms. In addition to its canonical role in glycolysis, PKM2 exhibits non-metabolic (“moonlighting”) functions that contribute to oncogenesis [49]. Beyond catalyzing the final step of glycolysis, PKM2 translocates to the nucleus, where it acts as a protein kinase to phosphorylate histones (e.g., H3T11) and transcription factors such as STAT3 and β-catenin, thereby promoting the transcription of genes involved in cell proliferation, survival, and hypoxia adaptation [50–53]. Nuclear PKM2 also interacts with hypoxia-inducible factor 1α (HIF-1α) to enhance the expression of glycolytic genes under low-oxygen conditions, creating a feedforward loop that sustains the Warburg effect [54]. These non-canonical roles enable PKM2 to coordinate metabolic reprogramming with transcriptional regulation, amplifying malignancy. Our observation that TACC3 silencing reduces PKM2 expression while increasing PKM1 raises the possibility that TACC3 may influence both the metabolic (glycolytic) and nuclear (kinase) functions of PKM2. Future studies should explore whether TACC3 modulates PKM2 splicing or subcellular localization, thereby regulating its dual roles in bladder cancer progression.

Recent studies have highlighted the critical role of c-Myc acetylation and deacetylation in modulating its stability and transcriptional activity. Here, we demonstrate that TACC3 promotes glycolysis in bladder cancer (BC) by interacting with c-Myc, enhancing its acetylation at lysine 323 (K323) and thereby stabilizing the c-Myc protein. Mechanistically, the physical interaction between TACC3 and c-Myc facilitates recruitment of the acetyltransferase PCAF while excluding the deacetylase SIRT1, creating an antagonistic balance that regulates c-Myc acetylation at K323. This acetylation stabilizes c-Myc by counteracting its ubiquitin-proteasome-mediated degradation, a process controlled by ubiquitin ligases (e.g., SKP2, FBW7, CHIP, FBXO32 [55–58]) and deubiquitinases (USP43, USP37, USP29 [59–61]). However, key questions remain unresolved, including whether acetylation directly competes with ubiquitylation at shared lysine residues or modulates interactions between c-Myc and ubiquitin ligases/deubiquitinases.

In summary, our findings indicate that E2F3 activates *TACC3* transcription, and TACC3 interacts with c-Myc to promote PCAF-mediated acetylation while excluding SIRT1, thereby preventing c-Myc deacetylation. Acetylated c-Myc exhibits increased stability and transcriptional activity, upregulating key glycolytic genes such as *GLUT1*, *HK2*, and *PFKFB3*. This TACC3-driven metabolic reprogramming facilitates abnormal glycolysis, supporting bladder cancer growth (Fig. 6N). Targeting the TACC3/c-Myc axis represents a promising therapeutic strategy to disrupt metabolic dependencies and impede BC progression.

## Methods

### Cell Culture

Bladder cancer cell lines, including SW780, HT1197, CAL29, 5637, TCCSUP, J82, UMUC10, and HEK293T embryonic kidney cells, were purchased from the American Type Culture Collection (ATCC). All cell lines were tested mycoplasma-free and maintained in Dulbecco’s Modified Eagle’s Medium (DMEM) supplemented with 10% fetal bovine serum (FBS) and 1% penicillin/streptomycin at 37 °C in a humidified atmosphere with 5% CO_2_. Cell identity was confirmed by short tandem repeat (STR) DNA profiling analysis.

### Transcription factor binding site prediction

Transcription factor binding site prediction was performed using the JASPAR database (available at https://jaspar.elixir.no/) [62].

### Western blotting and co-immunoprecipitation (co-IP)

For Western blotting, cells were collected and lysed in sample buffer (62.5 mM Tris-HCl, pH 6.8, 2% SDS, 25% glycerol). Equal amounts of total protein were resolved by 10% SDS-PAGE and transferred to polyvinylidene difluoride (PVDF) membranes. The membranes were blocked with 5% non-fat milk for 30 min at room temperature and then incubated with the indicated primary antibodies overnight at 4 °C. After washing, the membranes were incubated with horseradish peroxidase-conjugated secondary antibodies for 40 min at room temperature. Protein signals were detected using a chemiluminescence imaging instrument (GE LAS500).

For Co-IP assays, cells were lysed in IP buffer (150 mM NaCl, 5 mM EDTA, 0.5% NP-40, 50 mM Tris-HCl, pH 8.0) supplemented with PMSF (Sigma, USA) and a protease inhibitor cocktail. Cell lysates were centrifuged at 14,000 rpm for 12 min at 4 °C to remove debris. The clarified supernatants were incubated with the indicated antibodies, Flag-labeled beads, or GFP-labeled beads overnight at 4 °C. The immunoprecipitated beads were washed five times with IP buffer and subjected to SDS-PAGE, followed by immunoblotting.

### Plasmids and antibodies

The full-length coding sequence (CDS) region (amino acids 1–838), a specific domain (amino acids 639–838), and the N-terminal region (amino acids 1–638) of TACC3 were PCR-amplified and subsequently cloned into the pEGFP-C2 vector (EcoRI/BamHI sites). The full-length TACC3 was also cloned into the PLVX-puro vector. The CDS regions of human E2F3, MYC, and PCAF were amplified by PCR and cloned into the pcDNA3.1(+) vector with Flag or MYC tags.

The promoter regions of human TACC3 (−300/+300), GLUT1 (−400/+100), HK2 (−400/+100), and PFKFB3 (−400/+100) were amplified by PCR and cloned into the pGL3-Basic vector. Additionally, a synthetic promoter construct containing five tandem c-Myc response elements (E-box: CACGTG) was cloned into the pGL3-Basic vector. Site-directed mutagenesis was performed using the KODPlus Mutagenesis Kit (Toyobo) according to the manufacturer’s protocol.

The antibodies used in this study are listed below: Anti-TACC3 (Proteintech, 25697-1-AP), Anti-ACTB (Proteintech, 66009-1-Ig), Anti-E2F3 (Proteintech, 27615-1-AP), Anti-SIRT1 (Proteintech, 13161-1-AP), Anti-MYC (Cell Signaling Technology [CST], #9402), Anti-Max (S20) (CST, #4739), Anti-acetylated lysine (CST, #9441), Anti-PCAF (C14G9) (CST, #3378), Anti-MYC (9E10) (Santa Cruz, sc-40), Anti-GFP-tag (MBL, #598, 1:1000), Anti-DDDDK-tag (MBL, PM020, 1:1000).

### Transfection and lentiviral infection

Transient transfection of plasmids or siRNAs (Table S2) was performed using Lipofectamine 3000 according to the manufacturer’s protocol. To generate stable cell lines, HEK293T cells were transfected with overexpression plasmids or shRNA expression vectors, along with packaging plasmids (psPAX2 and pMD2G), for 48 h. The cell supernatant containing lentiviruses was collected, filtered through 0.45-μm filters (BIOFIL, Guangzhou, China), and used to infect target cells in the presence of 2 μg/mL polybrene (H9268, Sigma–Aldrich, St. Louis, MO, USA). After 24 h, infected cells were selected using 2 mg/mL puromycin (13884-500, Cayman Chemical, Ann Arbor, MI, USA).

### RNA extraction and quantitative PCR analysis

Total RNA was extracted using TRIzol reagent (Invitrogen). RNA concentration and quality were determined using a NanoDrop spectrophotometer (ND-1000, Thermo Scientific, USA). Total RNA was reverse-transcribed into cDNA using a cDNA Synthesis Kit (Thermo, K1622). The mRNA expression levels of the indicated genes were quantified using iQ™ SYBR Green Supermix (Bio-Rad, CA, USA) and a CFX96 real-time PCR detection system (Bio-Rad, CA, USA). ACTB was used as the internal control for normalization. The sequences of the amplification primers are listed in Table S3.

### RNA sequencing

Total RNA was extracted from 5637 cells with stable TACC3 knockdown and control cells using TRIzol reagent. Poly(A) RNA was isolated from total RNA using oligo(dT) magnetic beads (New England Biolabs). Purified poly(A) RNA samples were fragmented and reverse-transcribed into cDNA using random hexamer primers. The cDNA fragments were ligated to Illumina PE adapters, purified, and subjected to quality control using a Bioanalyzer 2100. Sequencing was performed using the HiSeq X Ten system (Illumina). Sequencing reads were aligned to the human reference genome (hg38) and normalized into FPKM (fragments per kilobase of transcript per million mapped reads) values (FPKM ≥0.1).

### BioGRID analysis

The physical interactors of TACC3 and c-Myc were identified using the BioGRID database (version 4.4.230, accessed January 2024). For TACC3 (UniProt ID: Q9Y6A5), all experimentally validated interactions (filtered for Homo sapiens) were extracted, excluding interactions with non-specific bait proteins. Similarly, interactions for c-Myc (UniProt ID: P01106) were retrieved using the same criteria. Overlapping interactors between TACC3 and c-Myc were identified by comparing the two lists via intersection analysis. Only proteins confirmed by low-throughput studies (e.g., co-immunoprecipitation, affinity capture-WB) were considered high-confidence interactors.

### Immunofluorescence analysis

Bladder cancer cells (1 × 10^4^) were plated on coverslips. After 30 h, the coverslips with cells were fixed with 4% paraformaldehyde for 15 min, permeabilized with 0.1% Triton X-100 for 5 min, and blocked with 5% bovine serum albumin (BSA) in phosphate-buffered saline (PBS) for 30 min at room temperature. The cells were then incubated with the indicated primary antibodies overnight at 4 °C, followed by incubation with fluorophore-conjugated secondary antibodies for 40 min at room temperature. The coverslips were counterstained with 4’,6-diamidino-2-phenylindole (DAPI) for 5 min and imaged using a confocal laser scanning microscope (ZEISS, LSM900).

### Dual-luciferase assay

Stable cell lines at 30% confluence in 24-well plates were co-transfected with vectors expressing Renilla luciferase and the indicated luciferase reporters for 36 h. Firefly luciferase (FLuc) and Renilla luciferase (RLuc) activities were measured using the Dual-Luciferase Assay System (Promega) according to the manufacturer’s instructions. The relative luciferase activity was normalized to Renilla activity.

### ChIP-qPCR assays

Chromatin immunoprecipitation (ChIP) assays were performed using the ChIP Assay Kit (Upstate). Indicated cells were crosslinked with 1% formaldehyde for 15 min at 37 °C and quenched with 0.125 M glycine. DNA was immunoprecipitated from sonicated cell lysates by overnight incubation with the indicated antibodies, with IgG antibodies serving as negative controls. Chromatin supernatants were analyzed by qPCR as described above. Primer sequences for ChIP-qPCR targeted the promoter (−316/−191) and leader (+22/+145) regions of TACC3 and the promoter region of *GLUT1*, *HK2*, and *PFKFB3* are listed in Table S4.

### Glycolysis and mitochondrial respiration analysis

Glucose uptake and lactate release were measured using the Glucose Assay Kit and Lactate Assay Kit (Solarbio, China), respectively, according to the manufacturer’s protocols. Cells were washed, trypsinized, counted, and seeded onto an XFe96 cell culture microplate (Agilent Technologies, USA) for Seahorse analysis. Glycolytic and mitochondrial parameters were assessed using the XF Glycolysis Stress Test Kit (#103020-100, Seahorse Bioscience) and the Seahorse XFe96 Extracellular Flux Analyzer.

For glycolysis measurements, cells were equilibrated in a bicarbonate-free L-15 medium supplemented with 1 mM glutamine and incubated at 37 °C for 60 min to stabilize pH and temperature. Basal extracellular acidification rate (ECAR) was recorded, followed by sequential injections of 10 mM glucose, 1 µM oligomycin (Cayman Chemicals), and 50 mM 2-deoxy-d-glucose (2-DG, Sigma) to inhibit glycolysis. Mitochondrial respiration was analyzed by measuring oxygen consumption rate (OCR) under baseline conditions, followed by sequential additions of 2 µM oligomycin (ATP synthase inhibitor), 1 µM FCCP (carbonyl cyanide-4-(trifluoromethoxy)-phenylhydrazone, Cayman Chemical), and 0.5 µM rotenone/antimycin A (AdipoGen/Sigma) to inhibit mitochondrial complexes I and III. Basal OCR was calculated by subtracting residual OCR (post-rotenone/antimycin A) from pre-oligomycin OCR, while maximal OCR was derived by subtracting residual OCR from FCCP-stimulated OCR.

Glycolytic proton efflux rate (glycoPER) was quantified using the Seahorse XF Glycolytic Rate Assay (Agilent Technologies), which integrates OCR and ECAR measurements to distinguish glycolytic acidification from mitochondrial CO_2_-driven effects. Data were normalized to protein content and analyzed using Wave Desktop software (Agilent Technologies).

### Cell proliferation and colony formation assays

Cell proliferation was assessed using the Cell Counting Kit-8 (CCK-8) (MCE, USA) according to the manufacturer’s instructions. Cells (1 × 10³) with the indicated treatments were seeded in 96-well plates and analyzed on the indicated days following standard procedures.

For colony formation assays, cells were plated in dishes in triplicate and maintained with the indicated treatments for 10–15 days. Colonies were fixed with methanol for 20 min and stained with 0.5% crystal violet (Sigma–Aldrich, Missouri, USA) for 20 min. The number of colonies was counted and analyzed.

### Animal experiments

TACC3-stable overexpressing CAL29 cells (2 × 10^6^) or TACC3-stable knockdown 5637 cells (3 × 10^6^), along with their respective control cells, were resuspended in 100 µL of DMEM containing 20% Matrigel (BD Biosciences) and injected subcutaneously into 5-week-old female BALB/c nude mice (purchased from Beijing Vital River Laboratory Animal Technology Co., Ltd). Tumor size was measured every 3 days using a vernier caliper starting 7 days after injection, and mice were sacrificed at the final time point.

For 2-deoxy-d-glucose (2-DG) treatment experiments, tumor-bearing mice were injected intraperitoneally with 2-DG (600 mg/kg body weight) or saline as a control every other day. Tumor size was measured every 3 days using a vernier caliper, and mice were sacrificed at the final time point. Xenograft tumors were isolated, weighed, and processed for subsequent analysis.

### Statistical analysis

Statistical analyses were performed using GraphPad Prism 8.0 software. Student’s *t*-test was used to compare two independent groups, and one-way ANOVA with Tukey’s multiple comparisons test was used to compare three or more groups. Pearson correlation analysis and chi-square tests were used to examine correlations between continuous variables. A *P* value of less than 0.05 was considered statistically significant. All analyses were conducted using GraphPad Prism 8.0 software.

## Supplementary information

Supplementary data

Supplementary tables

WB unprocessed

## Data Availability

Primary datasets have been generated and would be deposited in the Research Data Deposit (RDD) public platform (http://www.researchdata. org.cn). All other data supporting the findings of this study are available from the corresponding author upon reasonable request.
