# Supplementary material for: TACC3 enhances glycolysis in bladder cancer cells through inducing acetylation of c-Myc
Source: Cell Death Dis. 2025 Apr 17;16(1):311. doi: 10.1038/s41419-025-07645-6 (PMC12006502; doi:10.1038/s41419-025-07645-6)
Supplement: Supplementary file 1 — Supplementary data [file 41419_2025_7645_MOESM1_ESM.docx]

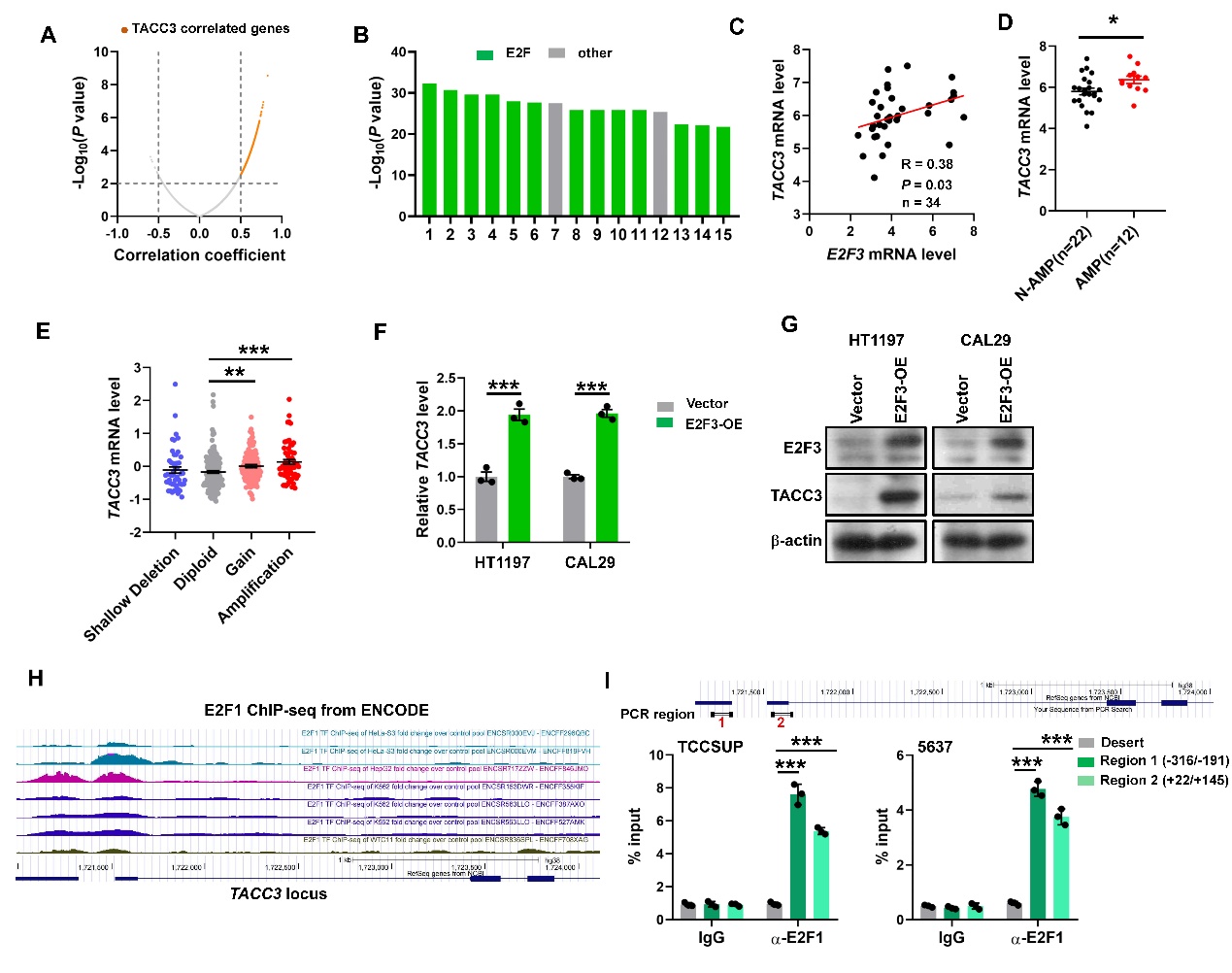


**Figure S1 TACC3 expression is associated with E2F3 amplification**

1. Scatterplot depicting the correlation between *TACC3* mRNA level versus other genes mRNA levels in 34 BC cell lines.
2. GSEA of TF factors that were enriched in the list of genes that are highly correlated with *TACC3* expression. The most enriched pathway is the E2F family transcription factors.
3. Scatterplot depicting the correlation between *E2F3* mRNA level and *TACC3* mRNA level. TPM stands for transcripts per million clean reads. Pearson correlation coefficient (r) and p value were indicated on the plot. Linear regression was represented by the red line.
4. Comparison of mRNA expression of *TACC3* between *E2F3* near-diploid and amplification BC cell lines.
5. Comparison of mRNA expression of *TACC3* among *E2F3* copy number status in TCGA-BC cohort.
6. The E2F3 and TACC3 mRNA levels in T24 and CAL29 cells transfected with E2F3 CDS plasmid or vector.
7. Western blot analysis of E2F3 and TACC3 in E2F3 OE plasmid-transfected T24 and CAL29 cells.
8. ENCODE ChIP-seq data mapping showing E2F1 enrichment at TACC3 promoter regions in HeLa-S3, HepG2, K562 and WTC11 cells.
9. Upper, Schematic diagram of PCR amplification region at TACC3 gene locus. Lower, ChIP-qPCR assays showing E2F3 binding at the promoter region (-316/-191) and leader region (+22/+145) of TACC3 in TCCSUP and 5637 cells. IgG serves as a negative control. Data shown means ± SD. Significance were calculated using one-way ANOVA (A) or two tailed student’s t test. *** P < 0.001.


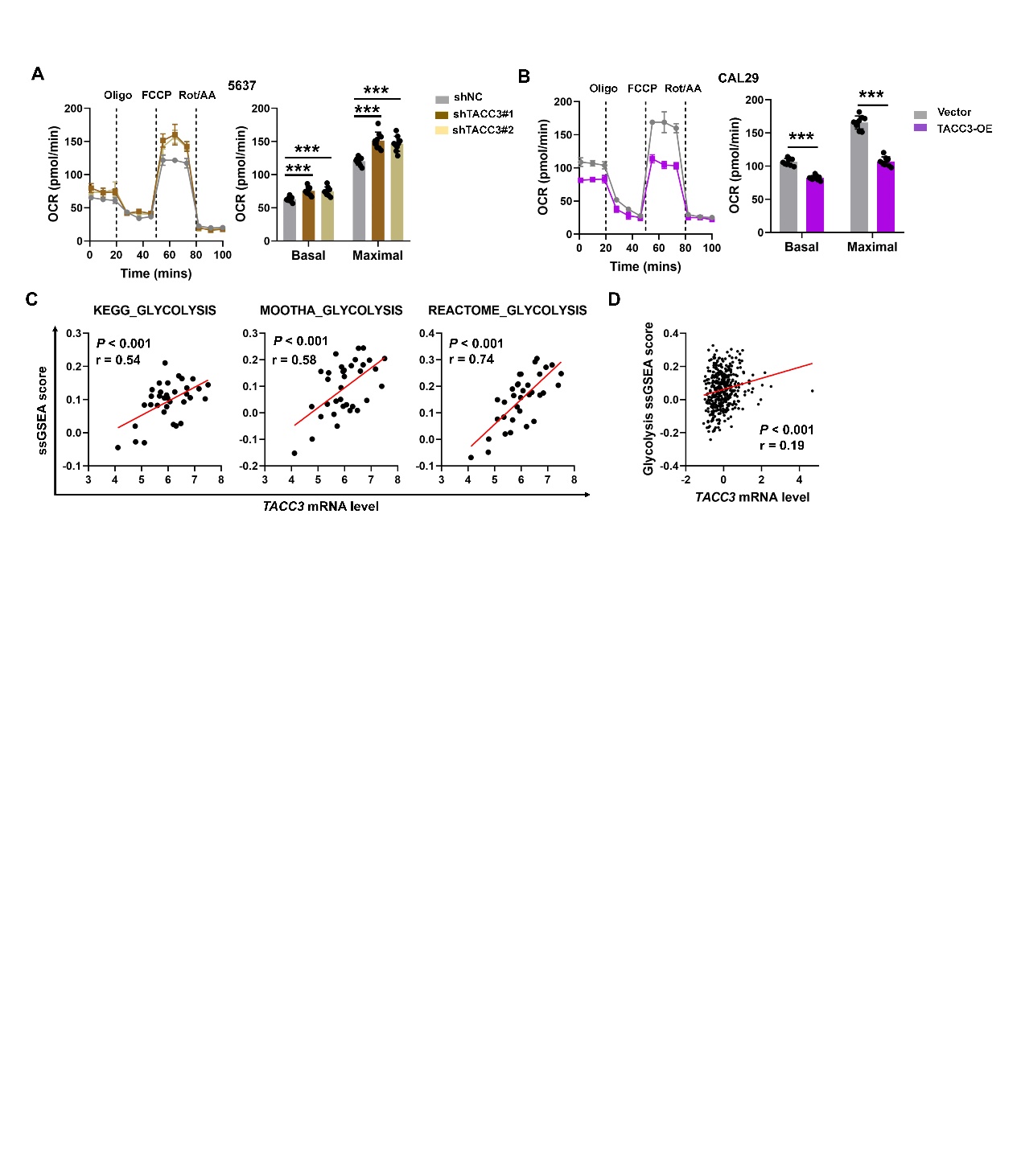


**Figure S2 TACC3 expression is associated with** **glycolysis score**

A-B. Analyzes of oxygen consumption rate (OCR) of TACC3 knockdown (A) or overexpressing (B) cells via a Seahorse extracellular flux analyzer. Oligo, oligomycin. FCCP, Carbonyl cyanide 4-(trifluoromethoxy)phenylhydrazone. Rot/AA, Rotenone/Antimycin A. Quantitative analysis of basal and maximal oxygen consumption rate in the right panels. Data show means ± SD. Significance were calculated using one-way ANOVA or two-tailed student’s t test, ****p* < 0.001.

1. Pearson analysis of the correlation between TACC3 mRNA levels with ssGSEA score of glycolysis in BC cell lines.
2. Pearson analysis of the correlation between the levels of TACC3 with ssGSEA score of glycolysis in the TCGA cohort.


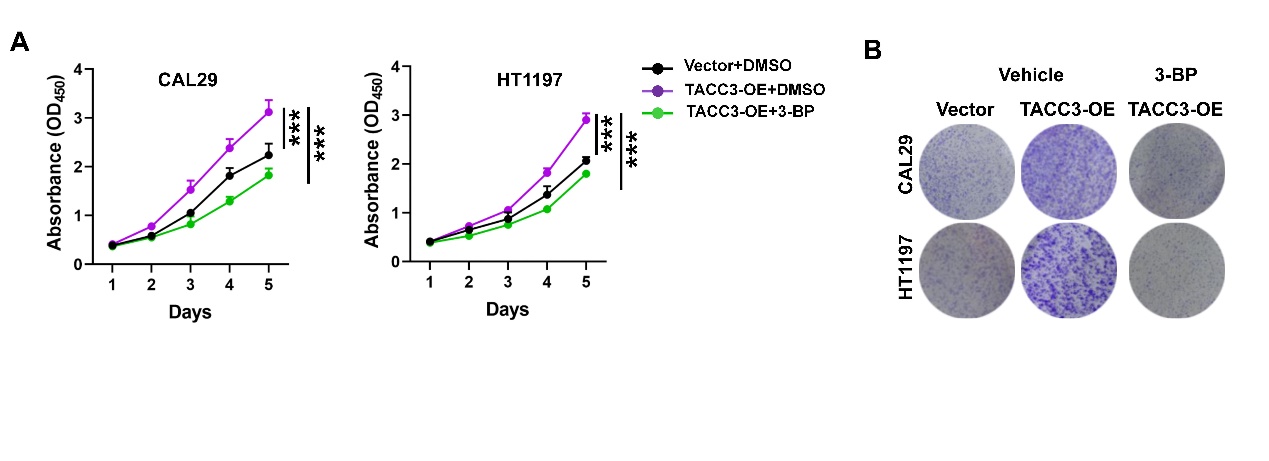


**Figure S3 Glycolysis inhibitor 3-BP reverses the promotion effect of TACC3 overexpression**

1. Cell proliferation in CAL29 or HT1197 cells with indicated treatment.
2. Colony formation assays in indicated cells with indicated treatment.

Experiments have repeated triplicate. 3-BP (15 ug/ml).

**
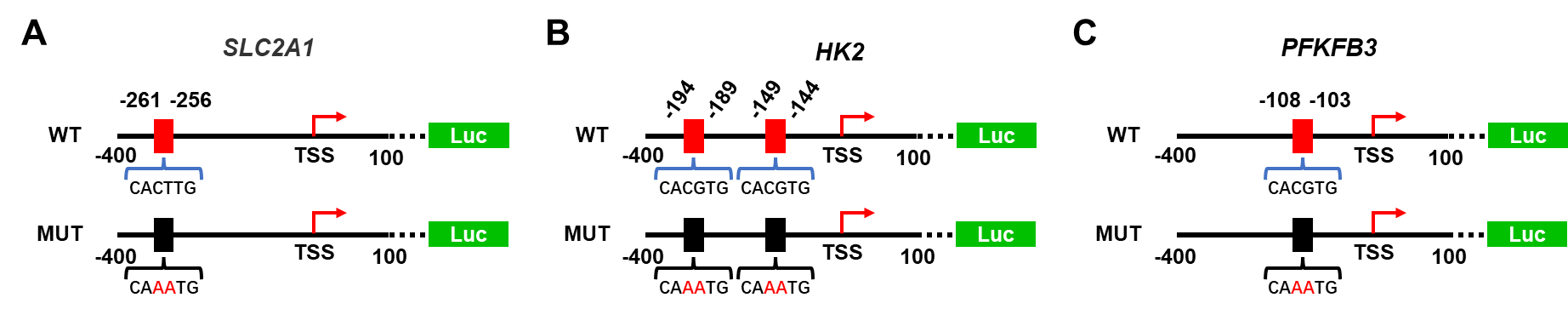
**

**Figure S4 Schematic diagram of the constructed luciferase reporters containing wild-type or mutant promoter sequences of indicated glycolysis genes.**

The transcription start site (TSS) is marked at position 0. The predicted c-Myc binding sites are located at indicated positions relative to the TSS. Wild-type (WT) and mutated (MUT) promoter fragments used in luciferase assays are depicted. Mutations are indicated by red Xs. SLC2A1 (A), HK2 (B), PFKFB3 (C).
