# Supplementary material for: TACC3 enhances glycolysis in bladder cancer cells through inducing acetylation of c-Myc
Source: Cell Death Dis. 2025 Apr 17;16(1):311. doi: 10.1038/s41419-025-07645-6 (PMC12006502; doi:10.1038/s41419-025-07645-6)
Supplement: Supplementary file 3 — WB unprocessed [file 41419_2025_7645_MOESM3_ESM.pdf]

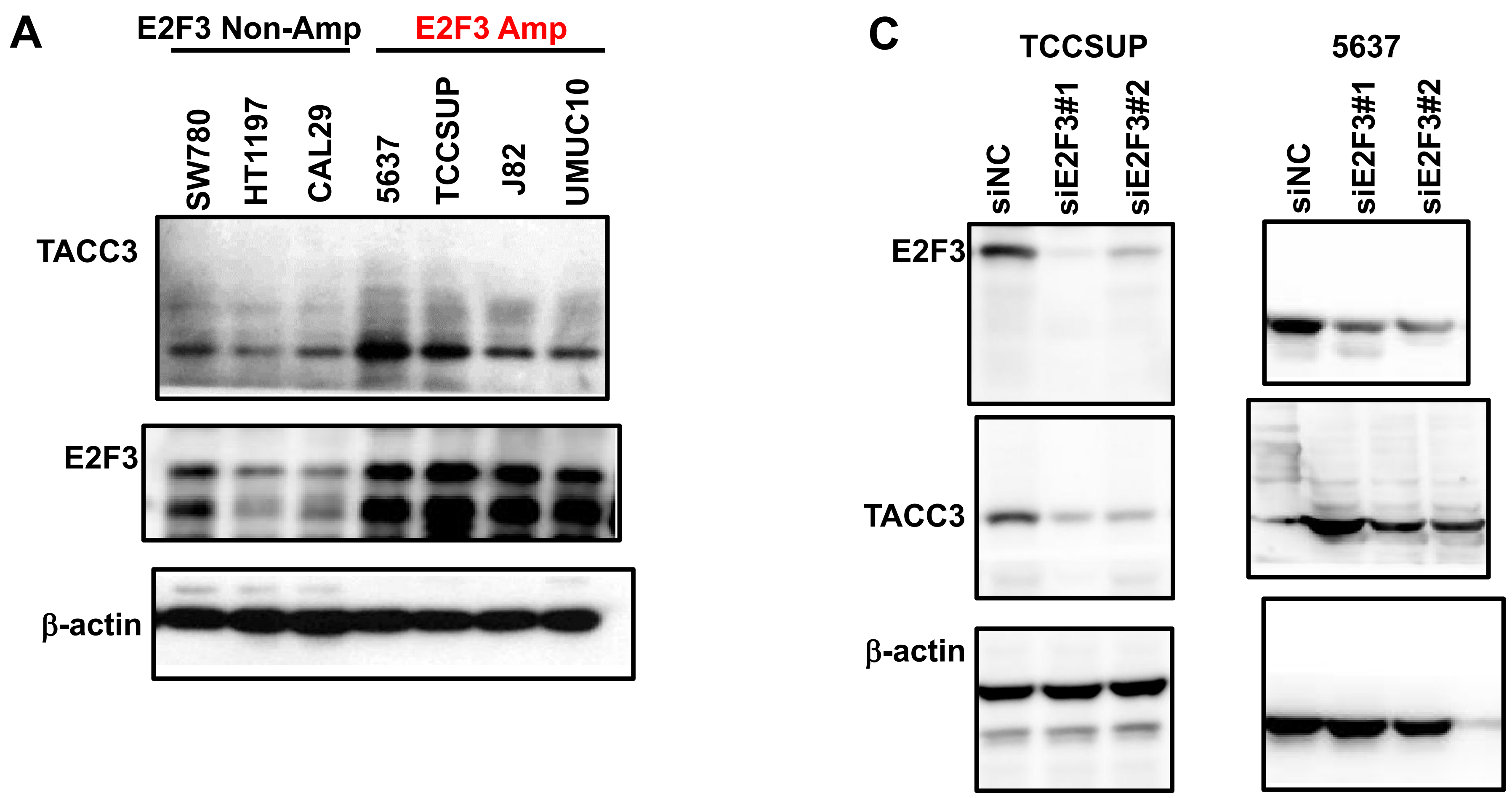
A
E2F3 Non-Amp
E2F3 Amp
SW780
HT1197
CAL29
5637
TCCSUP
J82
UMUC10
TACC3
E2F3
β-actin
C
TCCSUP
5637
siNC
siE2F3#1
siE2F3#2
siNC
siE2F3#1
siE2F3#2
E2F3
TACC3
β-actin

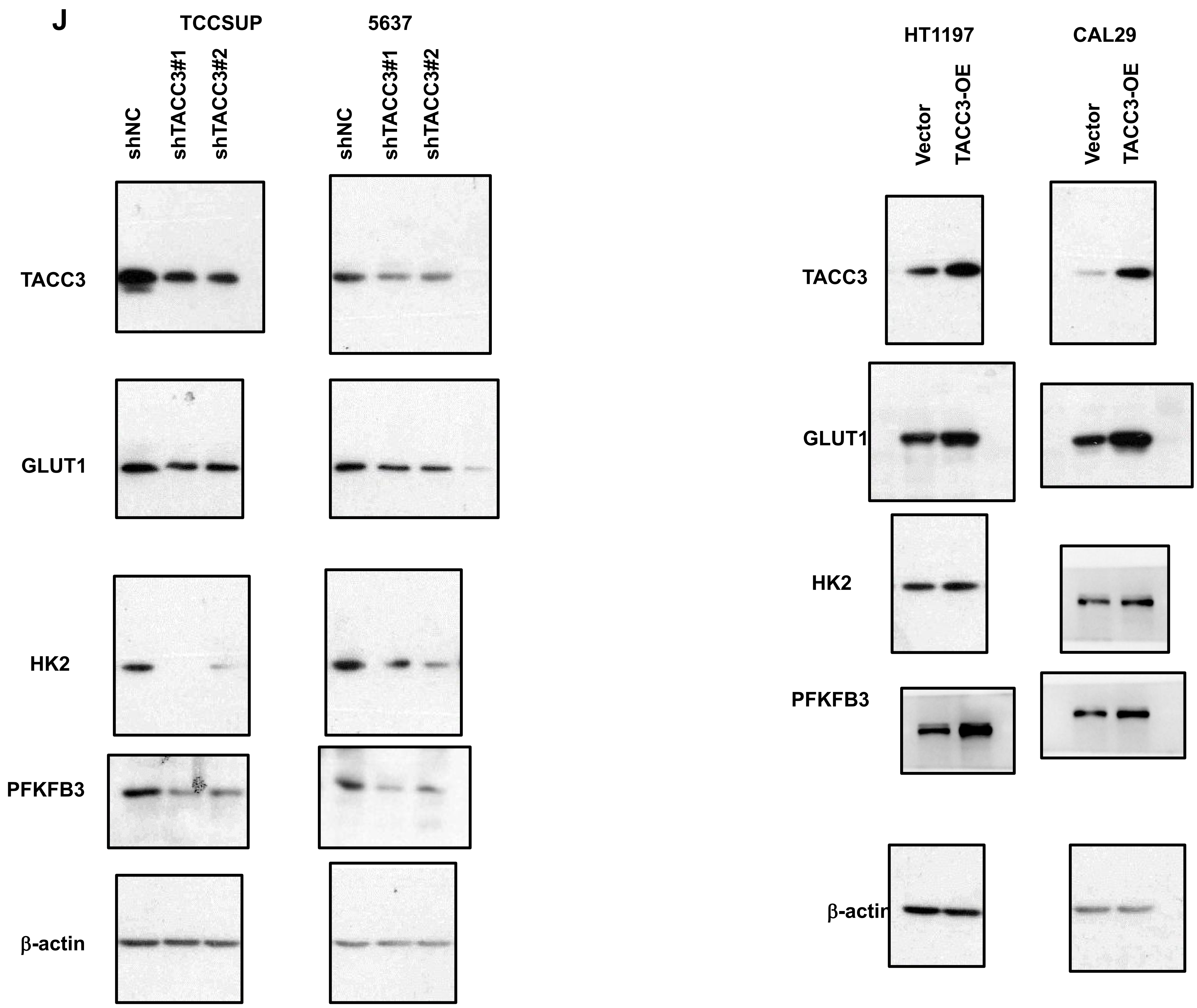
J
TCCSUP
5637
shNC
shTACC3#1
shTACC3#2
HT1197
CAL29
Vector
TACC3-OE
TACC3
GLUT1
HK2
PFKFB3
β-actin

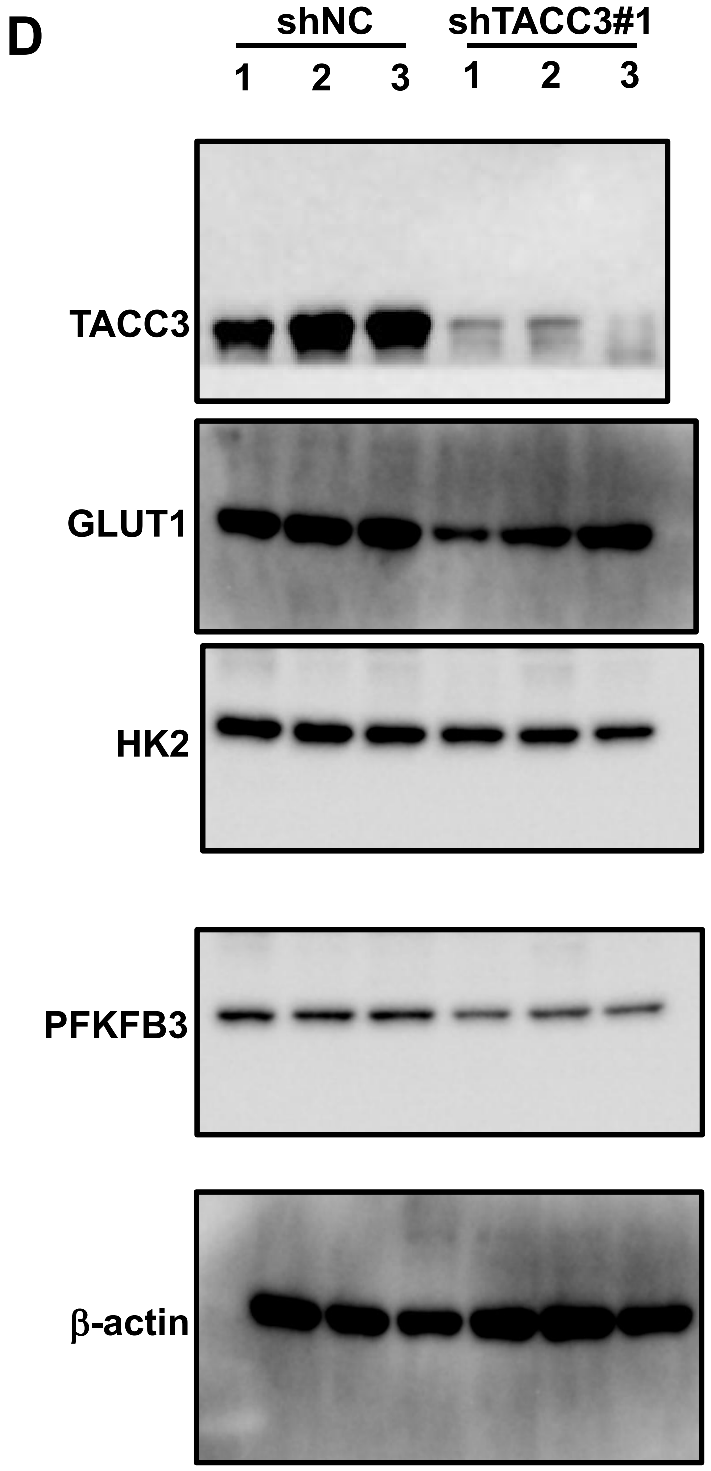
D
shNC
shTACC3#1
1 2 3 1 2 3
TACC3
GLUT1
HK2
PFKFB3
β-actin

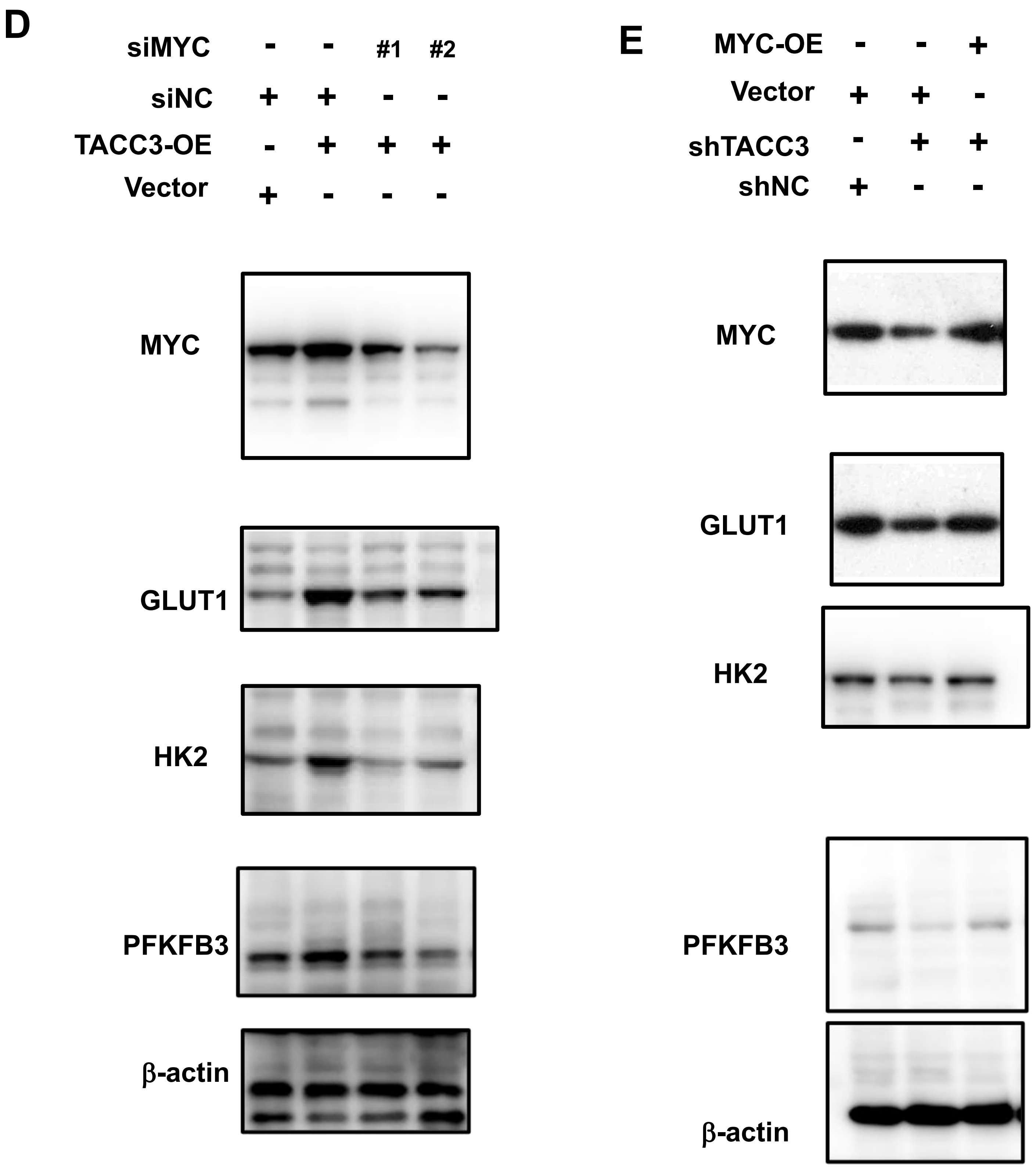
D
siMYC - - #1 #2
siNC + + - -
TACC3-OE - + + +
Vector + - - -
MYC
GLUT1
HK2
PFKFB3
β-actin
E
MYC-OE - - +
Vector + + -
shTACC3 - + +
shNC + - -
MYC
GLUT1
HK2
PFKFB3
β-actin

A

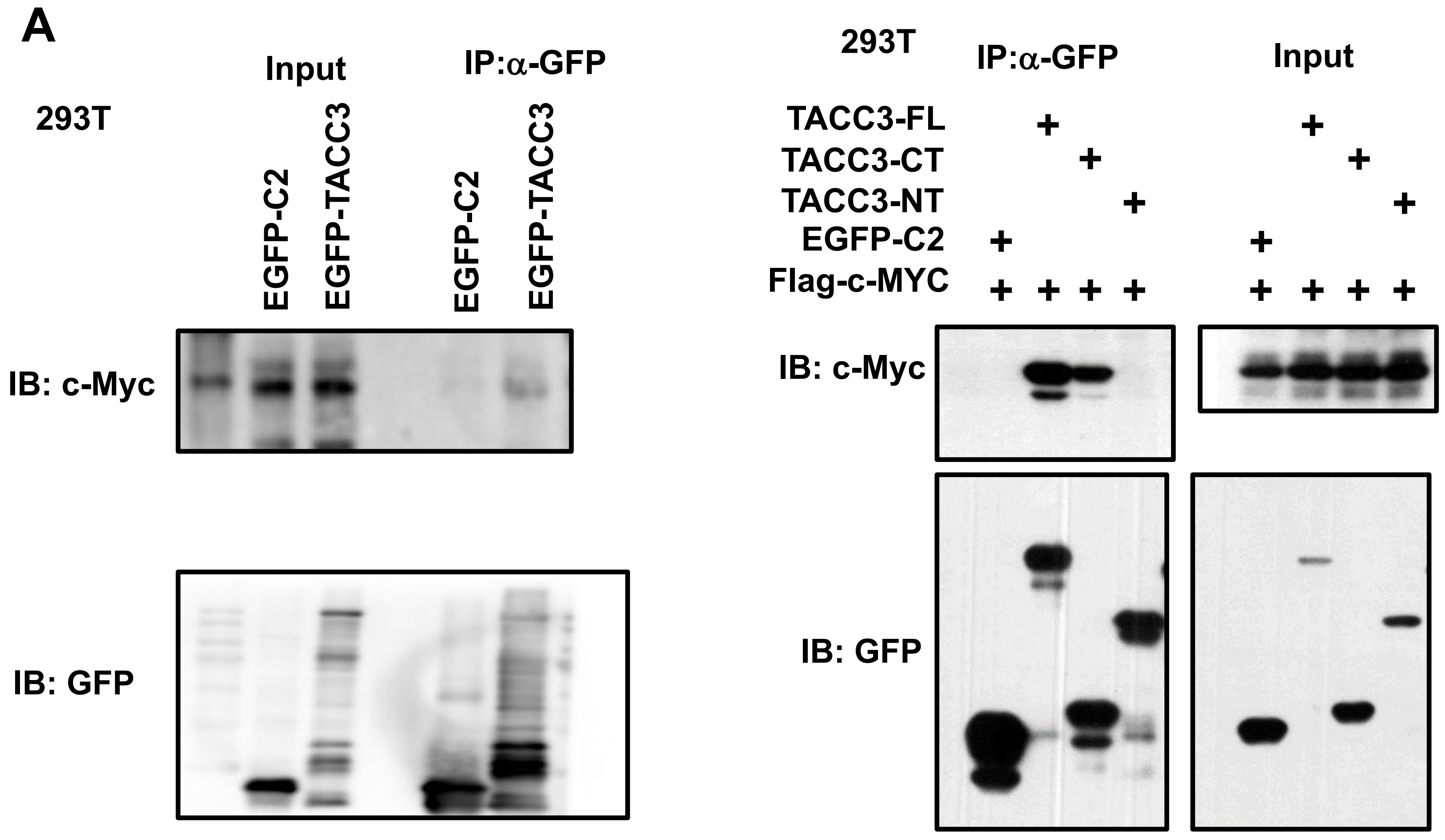
293T
Input
IP:α-GFP
EGFP-C2
EGFP-TACC3
EGFP-C2
EGFP-TACC3
IB: c-Myc
IB: GFP
293T
IP:α-GFP
Input
TACC3-FL
TACC3-CT
TACC3-NT
EGFP-C2
Flag-c-MYC
IB: c-Myc
IB: GFP

C

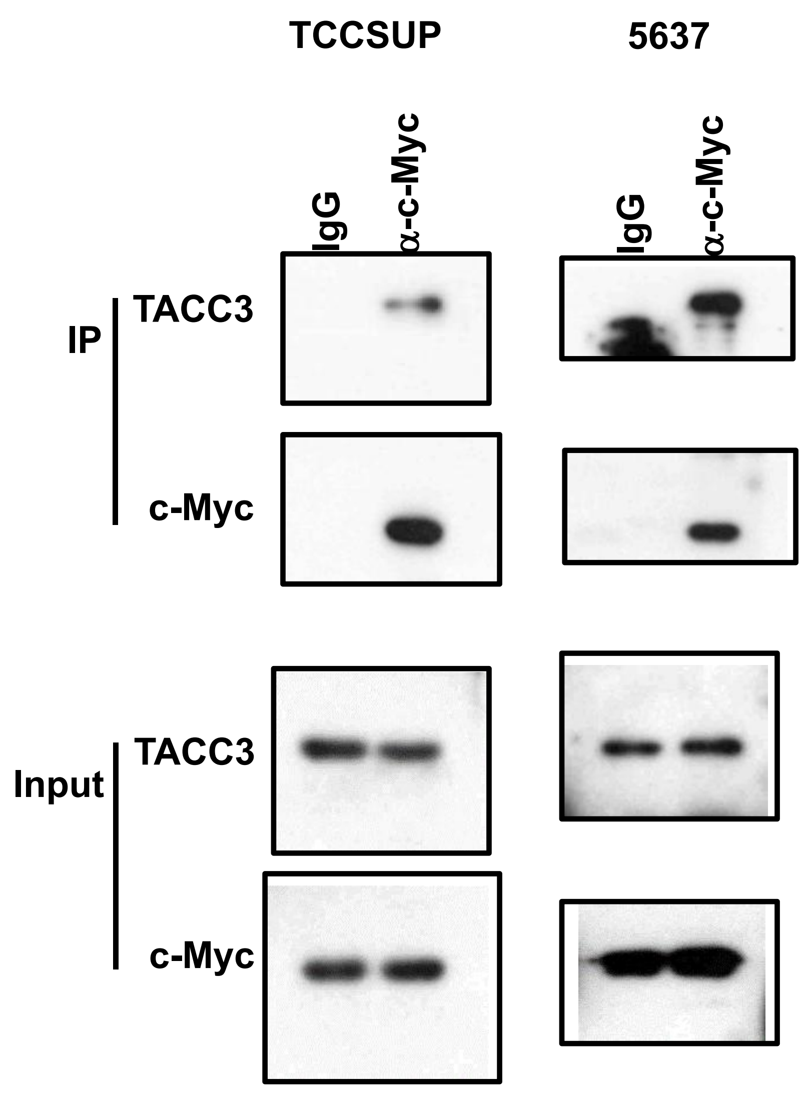
TCCSUP
5637
IgG
α-c-Myc
IgG
α-c-Myc
IP
TACC3
c-Myc
Input
TACC3
c-Myc

E

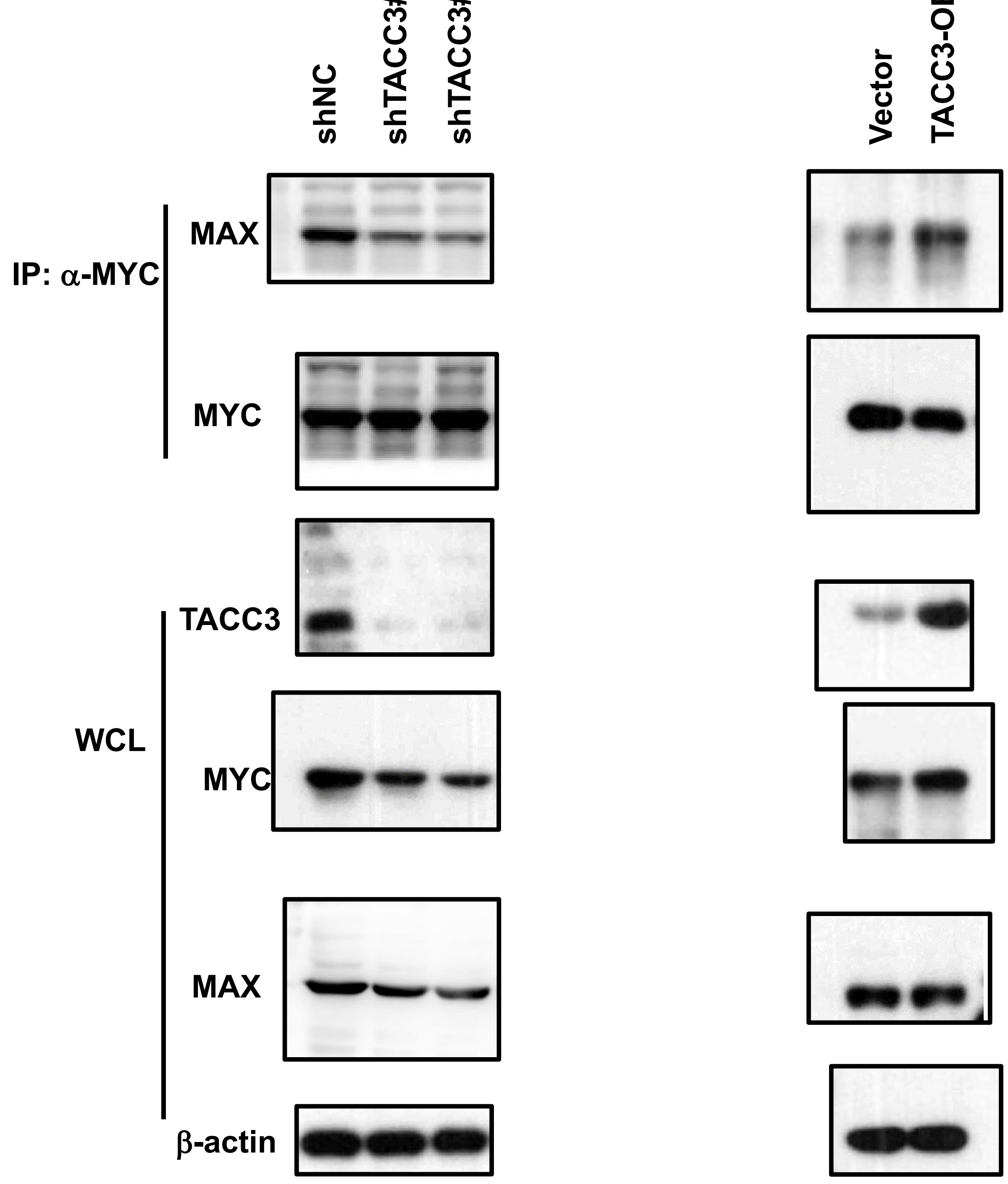
shNC
shTACC3#1
shTACC3#2
Vector
TACC3-OE
IP: α-MYC
MAX
MYC
TACC3
WCL
MYC
MAX
β-actin

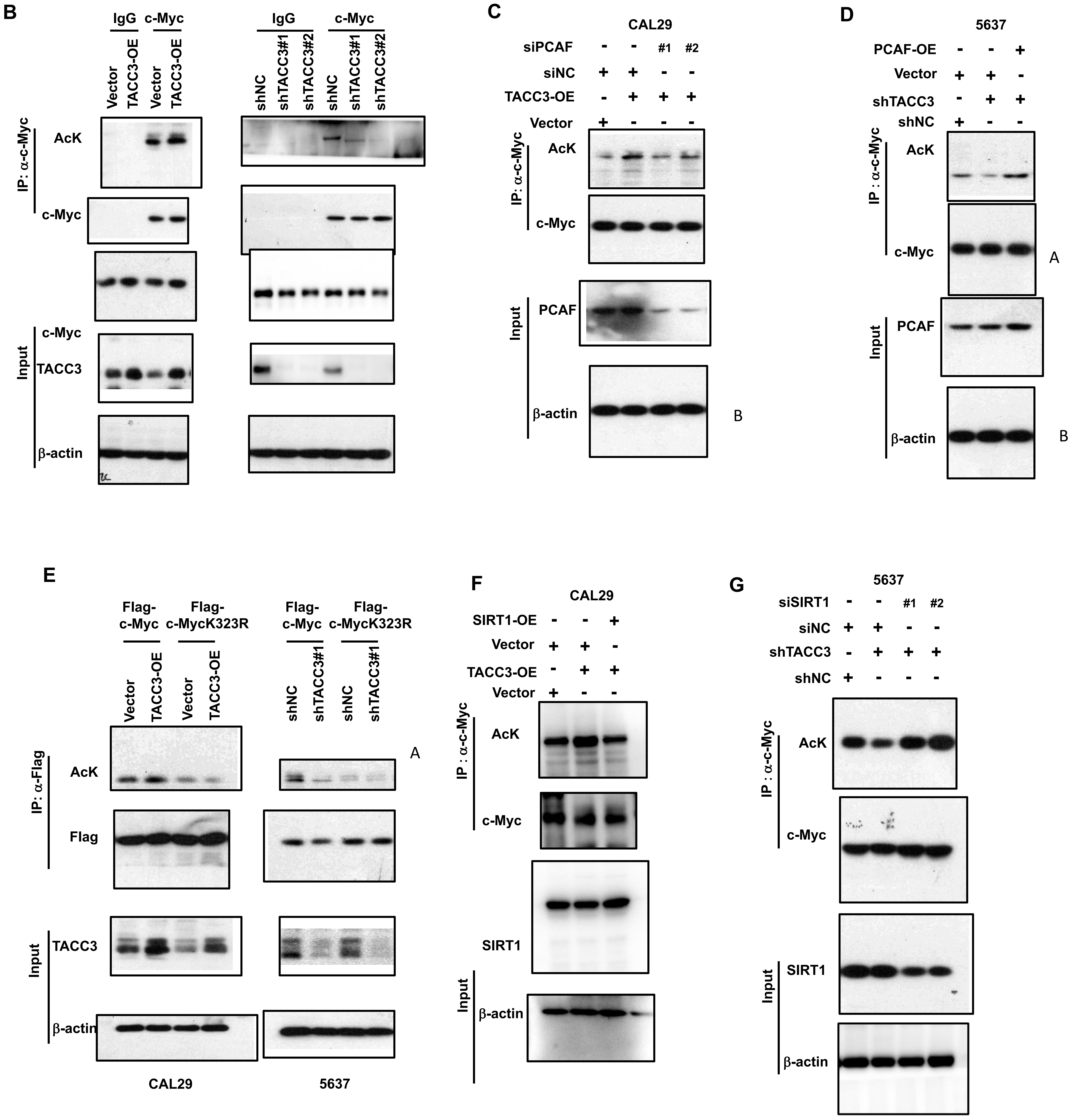
B
IgG
c-Myc
Vector
TACC3-OE
Vector
TACC3-OE
IgG
c-Myc
shNC
shTACC3#1
shTACC3#2
shNC
shTACC3#1
shTACC3#2
IP: α-c-Myc
AcK
c-Myc
Input
c-Myc
TACC3
β-actin
C
CAL29
siPCAF
- - #1 #2
siNC
+ + - -
TACC3-OE
- + + +
Vector
+ - - -
IP: α-c-Myc
AcK
c-Myc
Input
PCAF
β-actin
B
D
5637
PCAF-OE
- - +
Vector
+ + -
shTACC3
- + +
shNC
+ - -
IP : α-c-Myc
AcK
c-Myc
A
Input
PCAF
β-actin
B
E
Flag-
c-Myc
Flag-
c-MycK323R
Vector
TACC3-OE
Vector
TACC3-OE
Flag-
c-Myc
Flag-
c-MycK323R
shNC
shTACC3#1
shNC
shTACC3#1
A
IP: α-Flag
AcK
Flag
Input
TACC3
β-actin
CAL29
5637
F
CAL29
SIRT1-OE
- - +
Vector
+ + -
TACC3-OE
- + +
Vector
+ - -
IP : α-c-Myc
AcK
c-Myc
SIRT1
Input
β-actin
G
5637
siSIRT1
- - #1 #2
siNC
+ + - -
shTACC3
- + + +
shNC
+ - - -
IP : α-c-Myc
AcK
c-Myc
Input
SIRT1
β-actin

H

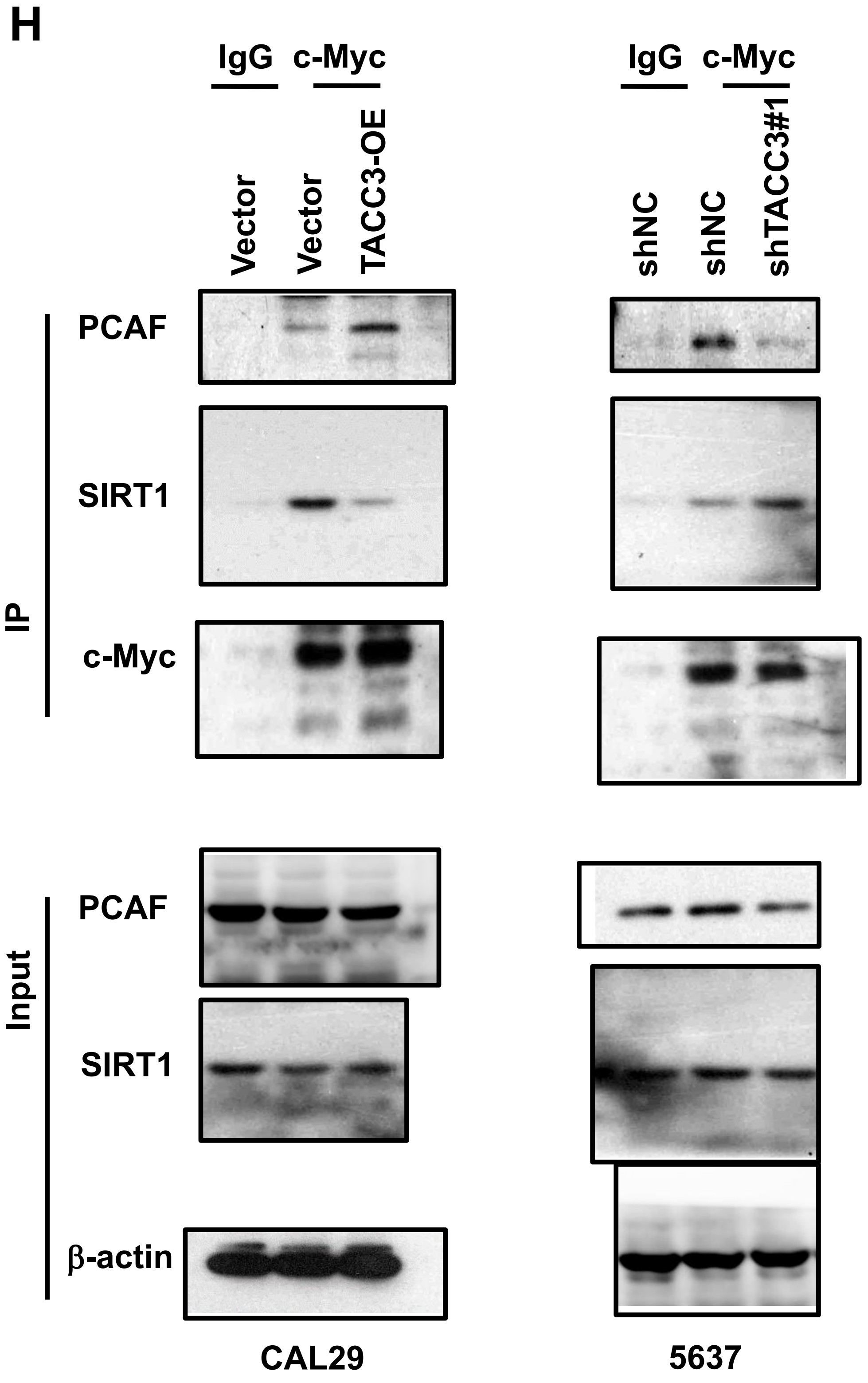
IgG
c-Myc
Vector
Vector
TACC3-OE
IgG
c-Myc
shNC
shNC
shTACC3#1
IP
PCAF
SIRT1
c-Myc
Input
PCAF
SIRT1
β-actin
CAL29
5637

I

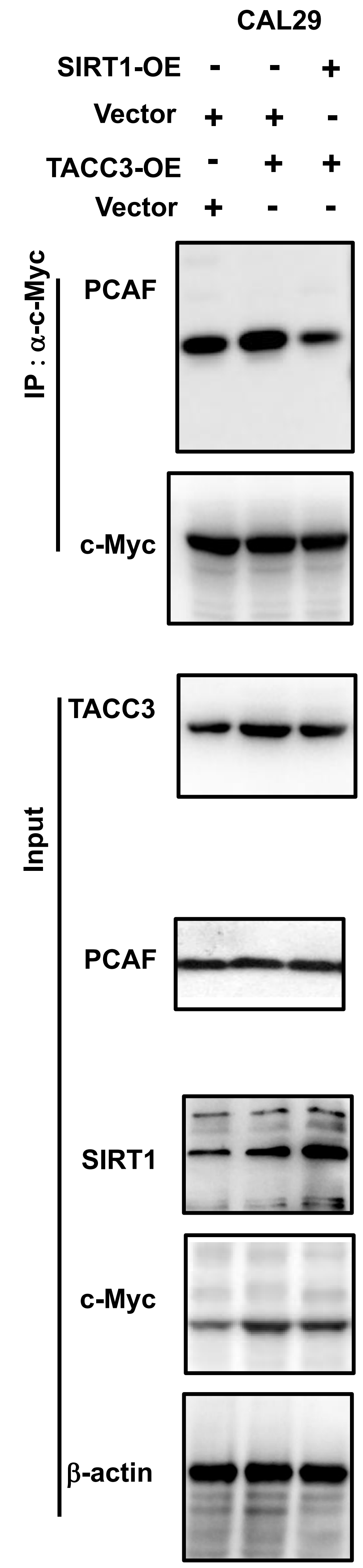
CAL29
SIRT1-OE - - +
Vector + + -
TACC3-OE - + +
Vector + - -
IP : α-c-Myc
PCAF
c-Myc
Input
TACC3
PCAF
SIRT1
c-Myc
β-actin

J

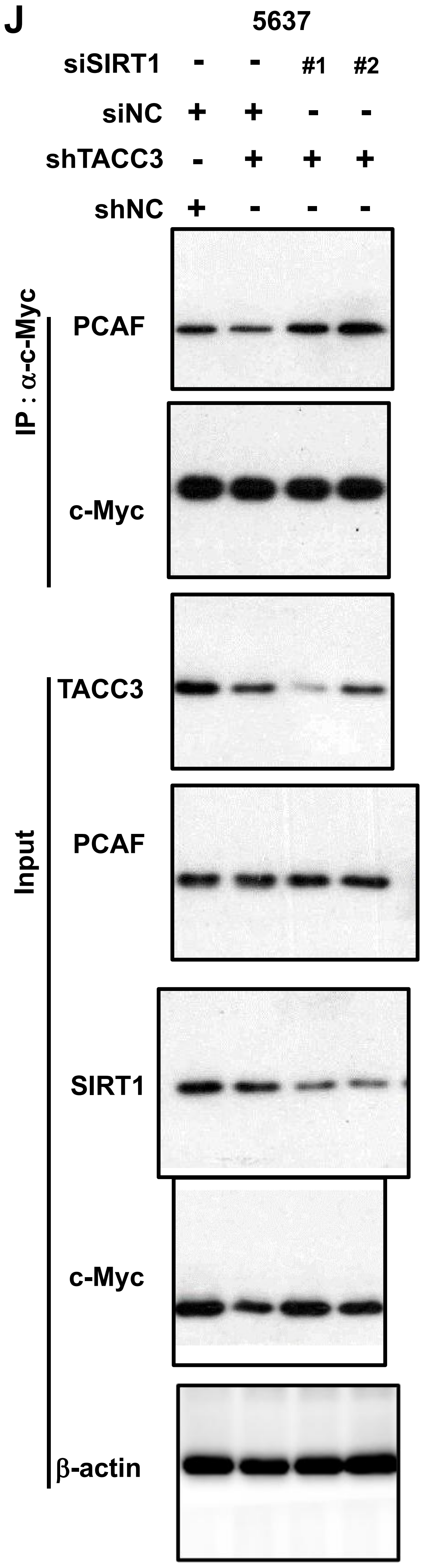
5637
siSIRT1 - - #1 #2
siNC + + - -
shTACC3 - + + +
shNC + - - -
IP : α-c-Myc
PCAF
c-Myc
Input
TACC3
PCAF
SIRT1
c-Myc
β-actin

K

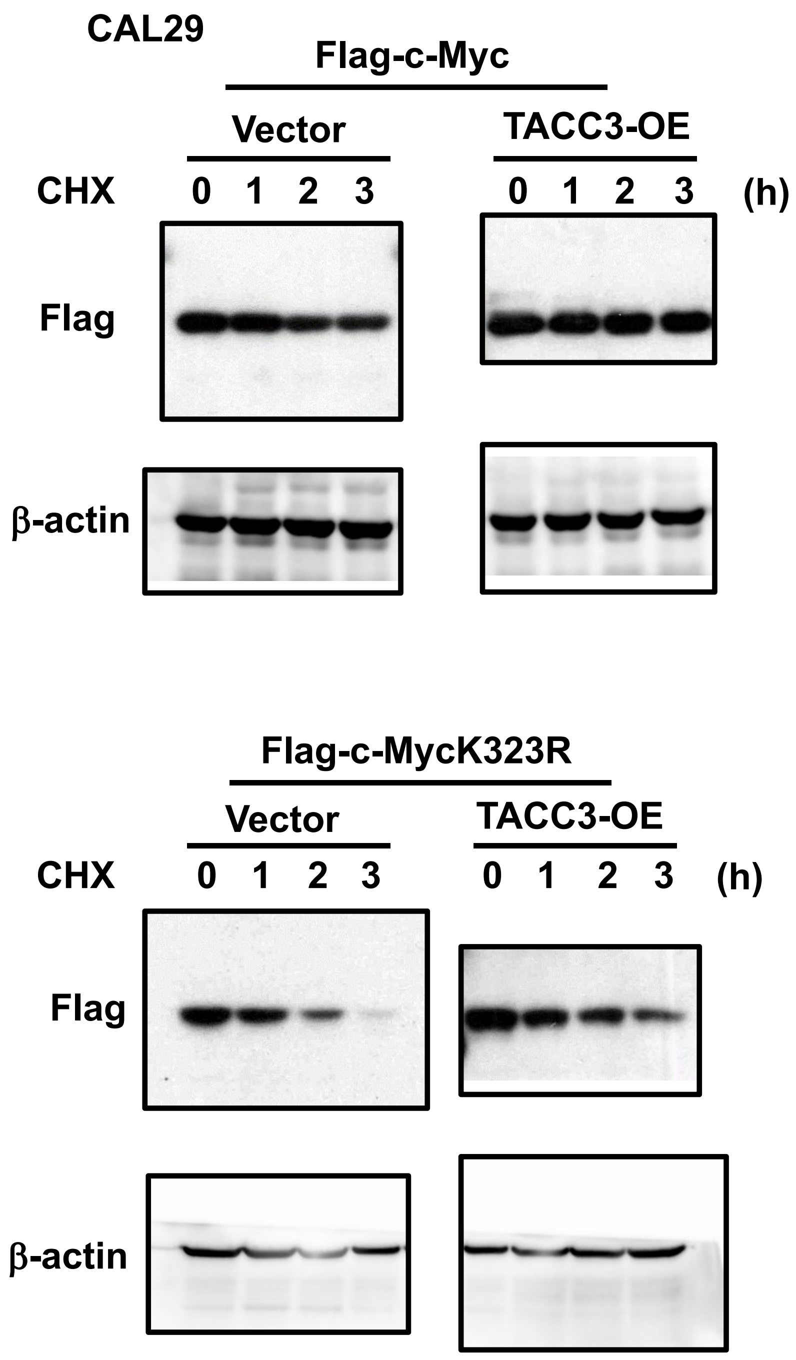
CAL29
Flag-c-Myc
Vector
TACC3-OE
CHX 0 1 2 3 0 1 2 3 (h)
Flag
β-actin
Flag-c-MycK323R
Vector
TACC3-OE
CHX 0 1 2 3 0 1 2 3 (h)
Flag
β-actin

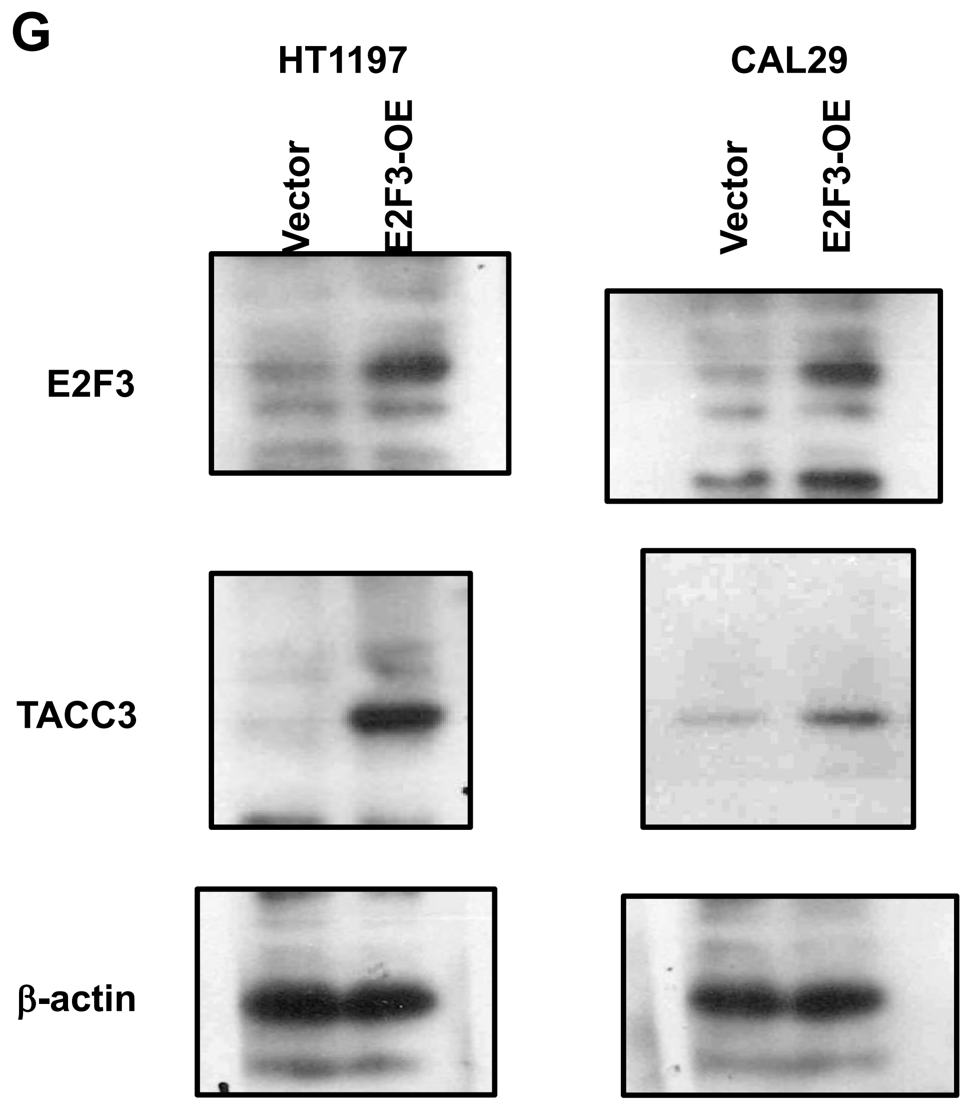
G
HT1197
CAL29
Vector
E2F3-OE
Vector
E2F3-OE
E2F3
TACC3
β-actin
